# Oxygen-rich microporous carbons with exceptional hydrogen storage capacity

**DOI:** 10.1038/s41467-017-01633-x

**Published:** 2017-11-16

**Authors:** L. Scott Blankenship, Norah Balahmar, Robert Mokaya

**Affiliations:** School of Chemistry, University of Nottingham, University Park, Nottingham, NG7 2RD UK

**Keywords:** Materials chemistry, Hydrogen storage materials, Porous materials

## Abstract

Porous carbons have been extensively investigated for hydrogen storage but, to date, appear to have an upper limit to their storage capacity. Here, in an effort to circumvent this upper limit, we explore the potential of oxygen-rich activated carbons. We describe cellulose acetate-derived carbons that combine high surface area (3800 m^2^ g^−1^) and pore volume (1.8 cm^3^ g^−1^) that arise almost entirely (>90%) from micropores, with an oxygen-rich nature. The carbons exhibit enhanced gravimetric hydrogen uptake (8.1 wt% total and 7.0 wt% excess) at −196 °C and 20 bar, rising to a total uptake of 8.9 wt% at 30 bar, and exceptional volumetric uptake of 44 g l^−1^ at 20 bar, and 48 g l^−1^ at 30 bar. At room temperature they store up to 0.8 wt% (excess) and 1.2 wt% (total) hydrogen at only 30 bar, and their isosteric heat of hydrogen adsorption is above 10 kJ mol^−1^.

## Abstract (web summary)

Hydrogen is attractive as a clean fuel for motor vehicles and porous carbons represent promising hydrogen storage materials. Here, Mokaya and colleagues incorporate oxygen-rich functional groups into porous carbons with high microporosity, showing that such materials exhibit significantly enhanced H_2_ storage capacity.

## Introduction

Hydrogen is attractive as a fuel to replace petrol in motor vehicles as part of the drive towards the anticipated hydrogen economy. Compared to currently used fossil fuels, hydrogen has advantages that make it more favourable as a fuel, including being potentially renewable and environmentally friendly (the sole by-product of hydrogen combustion or electrochemical oxidation is water, which would reduce CO_2_ emissions), and having a high gravimetric energy density^1–3^. However, the widespread storage, transportation and application of hydrogen as a practicable vehicular fuel is severely limited by technological obstacles related to the absence of viable hydrogen storage systems. For this reason, the storage of hydrogen in solid state materials is currently attracting a great deal of attention^4^. Materials that have high surface area such as porous carbons^5–12^, metal organic frameworks (MOFs)^13–17^ and covalent organic frameworks (COFs)^18,19^, have recently been the subject of intense study as potential stores for vehicular hydrogen storage. To date, studies suggest that the most important factors in determining adsorptive hydrogen uptake in porous materials are surface area, pore volume and pore size. In particular, many studies have shown a link between the surface area and amount of hydrogen stored in solid state materials^7–9,13–19^. It is now known that solid state materials that have micropores of size typically below 9 Å, combined with a high surface area, have the highest hydrogen storage levels^7–9,13–27^. The search for suitable hydrogen stores is typified by recent trends in MOFs and COFs wherein samples with surface area of up to 7000 m^2^ g^−1^ have been reported^16,17,28^. These high surface area MOFs and COFs are reported to have some of the highest hydrogen uptakes^16,17,28^. However, the amount of hydrogen stored in these and other high surface area materials is still severely limited by the weak interaction between molecular hydrogen and the surface of their pore walls. An ideal hydrogen storage material would have both high surface area and enhanced interaction with the adsorbed (i.e., stored) hydrogen molecules.

The weak interaction between adsorbed hydrogen and most porous materials can be improved via functionalisation of the surface of the pores. Porous carbons, in particular, lend themselves well to such functionalisation, and we and others have recently investigated the effect on hydrogen uptake of doping various heteroatoms onto carbon^29–35^. A question that has not been investigated is the effect of the level of oxygen content in porous carbons on hydrogen storage. This is a difficult question to investigate because carbons generally tend to contain a significant amount of oxygen. To date, no proper attention has been paid to the likely effects of the presence of elevated amounts of oxygen on the hydrogen storage capacity of porous carbons. Several studies have, however, hinted at positive effects on atomic hydrogen storage emanating from the presence of certain oxygen-containing functional groups on the surface of carbons^36–39^. For example, Yang et al.^36–38^ reported that the hydrogen storage capacity of metal-doped carbons could be significantly enhanced by introducing surface oxygen functional groups. It has also recently been reported that O-doping improves hydrogen storage capacity of pillared graphene boron nitride^39^. Although these previous studies are mainly related to adsorption of atomic hydrogen, it is of interest to experimentally probe the effect of elevated levels of oxygen content on physisorption-based hydrogen storage capacity of highly porous carbons.

Of greater relevance, therefore, to the present work are studies that have sought to determine the effect of oxygen content on the molecular hydrogen uptake of carbons, and which however offer contradictory conclusions^40–45^. The findings of Agarwal et al.^40^, that the hydrogen uptake of carbons increases with oxygen content are at odds with the claims of Huang et al.^41^, that for oxidised carbon surfaces, the hydrogen uptake reduces for higher levels of oxygen-containing functional groups. Schimmel et al.^42^, also concluded that the presence of oxygen exerts a negative influence on hydrogen uptake due to reduction in the amount of accessible aromatic C–C bonds. On the other hand, Llorens and Pera-Titus^43^ found that the presence of oxygen functional groups had no effect on the surface heterogeneity of carbons and thus did not influence the hydrogen–carbon sorption interaction. However, it is worth noting that, notwithstanding the conflicting conclusions, these previous studies were performed on carbon materials where both the oxygen content and textural properties varied such that increase in oxygen content effected changes to surface area, pore size and pore volume. Indeed, a theoretical study by Georgakis et al.^44^. concluded that the presence of oxygen functional groups within the pore channels can cause steric hindrances that limit the space available for storage of hydrogen. Similarly, Takagi et al.^45^ concluded that oxygen-containing functional groups can block the micropores that are responsible for hydrogen uptake. Variability in both oxygen content and pore size is not ideal for studies aimed at determining the effect of the former. Given the well-established critical role played by pore size in determining H_2_ uptake, one way to eliminate any ambiguities about the effect of oxygen content is to experimentally determine how the amount of oxygen affects H_2_ uptake in carbons with similar pore size or porosity. As far as we know, there have been no such studies that probe the effect of oxygen content in a set of carbon materials with identical or closely matched pore size. Perhaps the most rigorous study to date is that of Tellez-Juarez et al.^46^. However, the oxygen content range in that study was 2–11 wt%, which is typical for activated carbons, which means that the upper limit of oxygen content was too low to properly probe the effect of oxygen-rich carbon surfaces.

In this study, therefore, we exploited the relationship between the type of carbon precursor and nature of final activated carbon to generate a set of porous carbons that simultaneously combine high surface area and high level of microporosity along with a high oxygen content but have similar pore size. We investigated the characteristics of the oxygen-rich carbons and their hydrogen uptake properties and compared them to current benchmark porous carbon and MOF materials.

## Results

### Elemental composition and nature of carbon

In order to probe the effect of surface oxygen on the hydrogen uptake properties of porous carbons, we designed our carbon synthesis methods towards generating materials with a high oxygen content. For this reason, we used cellulose acetate, which is designated as CA (and has O/C atomic ratio of 0.93) as starting material rather than, for example, cellulose which has an O/C ratio of 0.83. Previous reports have shown that the oxygen content of biomass reduces following hydrothermal carbonisation (HTC) to hydrochar with the decrease being greater at higher HTC temperature^47–49^. The oxygen content reduces further when the hydrochar is activated with KOH with the extent of the decrease being greatest at higher activation temperature^5,7^. We therefore performed HTC of cellulose acetate at 250 °C in order to maximise the carbon yield whilst retaining a significant amount of oxygen. As shown in Table 1, the atomic O/C ratio of the resulting hydrochar (designated as CA-hydrochar) was 0.339 compared to 0.93 for cellulose acetate. However, it is noteworthy that the O/C ratio of the CA-hydrochar (0.339) is higher than a ratio of 0.263 for hydrochar derived from cellulose (designated as C-hydrochar) under similar HTC conditions (Supplementary Table 1)^7^. Table 1 also shows the elemental composition of the activated carbons obtained from the CA-hydrochar (designated as CA-4T carbons, where 4 is the KOH/hydrochar ratio and *T* is activation temperature in °C). The values given in Table 1 are the average of three measurements. As expected the elemental C content increases on activation of the CA-hydrochar but the increase is somewhat lower than what is typically observed for activation of biomass derived hydrochar^5,7^, with the consequence that the oxygen content remains high in the range of 18–23 wt% compared to ~30 wt% for the CA-hydrochar. This means that the O/C atomic ratio reduces from 0.339 for the CA-hydrochar to 0.225 (CA-4600), 0.165 (CA-4700) and 0.197 (CA-4800). The oxygen content of the cellulose acetate-derived CA-4T activated carbons is much higher than that of cellulose-derived carbons (Supplementary Table 1) prepared in a comparable manner^7^. (The cellulose acetate-derived CA-4T carbons were prepared under similar conditions to the C-4T carbons from cellulose except that the activation time of the former was 2 h compared to 1 h for the latter. We have used the C-4T samples as a baseline, and to highlight the extent to which differences in the final activated carbon depend on the choice of precursor; cellulose or cellulose acetate). The oxygen content of the cellulose-derived carbons ranges between 4.4 and 11.5 wt% compared to 18–23 wt% for the CA-4T samples (Supplementary Table 1). Thus the O/C ratio of cellulose-derived C-4T carbons is also much lower at 0.035–0.1 compared to 0.165–0.225 for the CA-4T samples. The elemental analysis data therefore show that the use of cellulose acetate-derived hydrochar as precursor generates activated carbons with much higher oxygen content compared to the use of cellulose-derived hydrochar. We note that direct activation of cellulose acetate was unsuccessful as no yield was obtained under our activation conditions.

**Table 1:** Elemental analysis of cellulose acetate-derived CA-hydrochar and activated carbons derived from the CA-hydrochar

Sample	C (%)	H (%)	O (%)	(O/C)^a^	(H/C)^a^
CA-hydrochar	66.2	3.9	29.9	0.339	0.707
CA-4600	76.1	1.1	22.8	0.225	0.173
CA-4700	81.4	0.7	17.9	0.165	0.103
CA-4800	78.3	1.1	20.6	0.197	0.169

^a^Atomic ratio

**Fig. 1:**
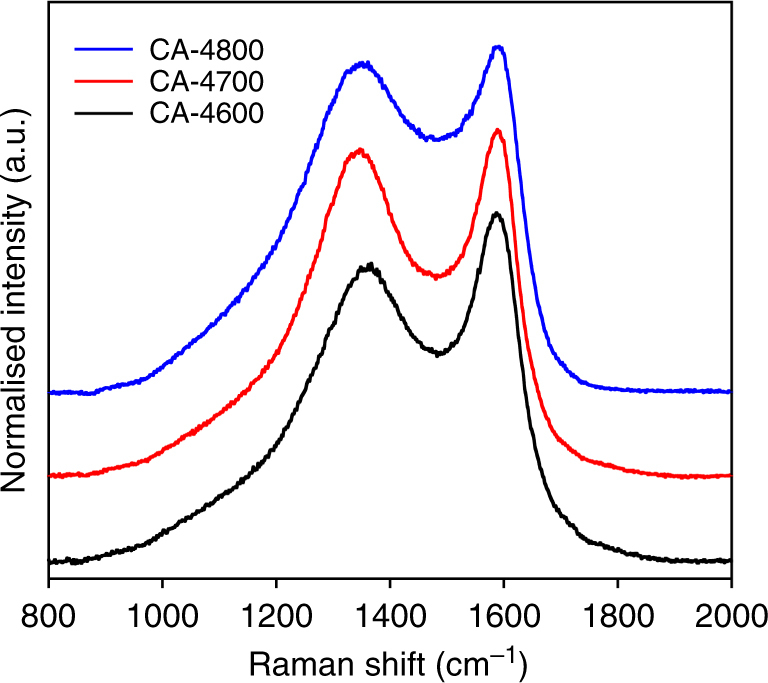
Raman spectra of activated carbons prepared from cellulose acetate. The activated carbons are labelled as CA-4T, where 4 is the activation KOH/carbon ratio and *T* is the activation temperature in °C

We confirmed that both the CA-hydrochar and CA-4T carbons were entirely carbonaceous by performing thermogravimetric analysis (TGA) in air; the samples were burnt up with virtually no residual mass at 700 °C (Supplementary Fig. 1). Similar to previous reports, the activated carbons have higher thermal stability compared to the hydrochar^50–52^. The CA-hydrochar has a burn off maximum of between 300 and 450 °C, while for the CA-4800 activated carbon the burn off maximum is at ca. 550 °C (Supplementary Fig. 1). The powder XRD pattern of the CA-hydrochar (Supplementary Fig. 2) shows a very broad feature centred at 2*θ* ≈ 22°, which is characteristic of non-graphitic carbon. Likewise the XRD patterns for the CA-4T-activated carbons (Supplementary Fig. 2) are relatively featureless, which indicates that the nature of the carbon in the samples is amorphous with few, if any, graphitic domains. That the CA-4T carbons are not graphitic is also evidenced by the Raman spectra shown in Fig. 1, which exhibit bands at 1345–1355 cm^−1^ and 1582–1588 cm^−1^ from the D-peak (disordered carbon) and the G-peak (graphitic domains), respectively^53^. The ratio of peak intensity (i.e., area) of the D-peak to G-peak (*I*_D_/*I*_G_ ratio) is 0.85 for CA-4600, 0.94 for CA-4700 and 1.00 for CA-4800. The *I*_D_/*I*_G_ ratio of 0.85–1.00 is consistent with the non-graphitic nature of the carbons^53^. We ascribe the increase in the *I*_D_/*I*_G_ ratio for activated carbons prepared at higher temperature to greater disruption of graphitic domains as a result of increased level of activation. The carbon yield from cellulose acetate to the CA-hydrochar was ca. 41%, which is similar to what has previously been reported^7,48,49^. The yield of activated carbons from the hydrochar varied between 20% (at 700 and 800 °C) and 35% (at 600 °C), which is in agreement with previous studies^7,48,49^.

### Porosity

**Fig. 2:**
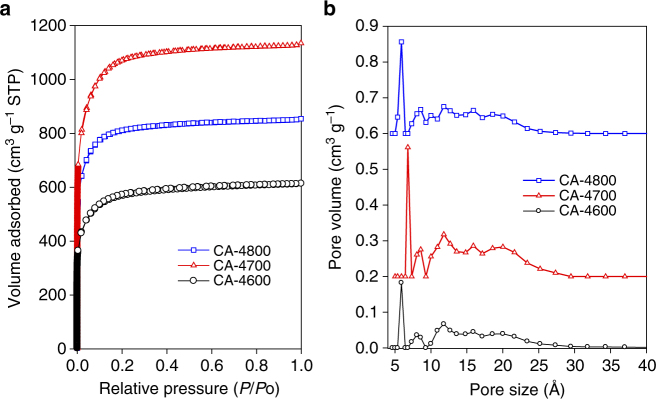
Porosity of activated carbons prepared from cellulose acetate. The activated carbons are labelled as CA-4T, where 4 is the activation KOH/carbon ratio and *T* is the activation temperature. The nitrogen sorption isotherms (**a**) and corresponding pore size distribution curves (**b**) are shown

The data discussed above confirm that the CA-4T carbons are oxygen rich with low levels of graphitisation. To be useful as hydrogen storage materials, the carbons also need to exhibit a high level of porosity that mainly arises from micropores^7–9,13–27^. The porosity of the carbons was probed using nitrogen sorption analysis. The nitrogen sorption isotherms of the activated carbons are shown in Fig. 2a. All three samples exhibit type I isotherms that are typically obtained for microporous materials^54^. The amount of nitrogen adsorbed, which is a measure of porosity in the carbons, rises significantly as activation temperature changes from 600 to 700 °C, and then decreases for the sample prepared at 800 °C. However, there is very little change in the shape of the isotherms suggesting that despite the large increase in porosity, the level of microporosity is maintained. Increase in overall porosity in activated carbons is normally associated with a change in the shape of the isotherm, and especially widening of the adsorption knee^5,7,55–60^.

The textural properties of the CA-4T carbons are summarised in Table 2. The apparent surface area (hereinafter referred to simply as surface area) rises from 2001 m^2^ g^−1^ for sample CA-4600 to a high of 3771 m^2^ g^−1^ for CA-4700 and then reduces to 2864 m^2^ g^−1^ for CA-4800. It is noteworthy that sample CA-4700 has one of the highest surface areas ever reported for activated carbons^24,55,57,61,62^. The pore volume follows a similar trend, rising from 0.95 cm^3^ g^−1^ for sample CA-4600 to 1.75 cm^3^ g^−1^ for CA-4700 and then reduces to 1.54 cm^3^ g^−1^ for CA-4800. What is remarkable is that despite their very high surface area and pore volume, the carbons exhibit a very high level of microporosity; for sample CA-4600, 90% of surface area arises from micropores, while for samples CA-4700 and CA-4800, the proportion of surface area from micropores is higher at 92%. The proportion of pore volume that arises from micropores is also very high at 83% for CA-4600 and 88% for both CA-4700 and CA-4800. It is particularly noteworthy that 92% (3484 m^2^ g^−1^) of the extremely high apparent surface area of 3771 m^2^ g^−1^ for sample CA-4700 arises from micropores. As far as we are aware, this is the highest micropore surface area (3484 m^2^ g^−1^) and proportion (92%) of area arising from micropores ever reported for an activated carbon^24,55,57,61,62^. We have previously reported on activated carbons that exhibit a high proportion of microporosity, but those samples tended to have lower levels of surface area (i.e., <2500 m^2^ g−1)^63^. The high level of microporosity in the CA-4T carbons is also evident from the pore size distribution curves in Fig. 2b. The pore size distribution for all three samples is similar; the carbons have mainly micropores consisting of ultramicropores of size 6–7 Å, small micropores (8.5 Å) and some medium-sized micropores centred at ca. 12 Å (Table 2). The carbons have only a small proportion of pores with size larger than 20 Å, which is consistent with their very high microporosity. The particle morphology of the CA-4T carbons is similar to that typically observed for activated carbons (Supplementary Fig. 3). The morphology is of particles with mainly smooth surfaces and large conchoidal cavities, which is consistent with what has previously been observed for hydrochar-derived activated carbons^5,7^. However, depending on the projection, sample CA-4700 does appear to have some particles with less smooth surfaces which may hint at either an effect of activation temperature on the morphology or a characteristic of it’s very high microporosity.

**Table 2:** Textural properties and H_2_ uptake of cellulose acetate-derived activated carbons

Sample	Surface area^a^ (m^2^ g^−1^)	Pore volume^b^ (cm^3^ g^−1^)	Pore size^c^ (Å)	H_2_ uptake^d,e^				
				(wt%)			(g l^−1^)^e^	
				1 bar	20 bar	30 bar	20 bar	30 bar
CA-4600	2001 (1790)	0.95 (0.79)	6/8.5/12	3.1 (3.1)	6.2 (5.6)	6.7 (5.8)	44 (40)	48 (41)
CA-4700	3771 (3484)	1.75 (1.54)	7/8.5/12	3.9 (3.9)	8.1 (7.0)	8.9 (7.2)	37 (32)	41 (33)
CA-4800	2864 (2662)	1.32 (1.17)	6/8.5/12	3.4 (3.4)	6.8 (6.0)	7.3 (6.1)	41 (34)	41 (34)

The values in the parenthesis refer to:

^a^micropore surface area

^b^micropore volume

^c^Pore size distribution maxima obtained from NLDFT analysis

^d^Gravimetric (wt%) and volumetric (g l^−1^) H_2_ uptake at −196 °C and various pressures (i.e., 1, 20 or 30 bar)

^e^The values in parenthesis are excess H_2_ uptake

We compared the porosity of the cellulose acetate-derived CA-4T carbons with that of similarly prepared cellulose-derived activated carbons (Supplementary Table 2). We first note that the trend in porosity as a function of activation temperature is similar for both sets of sample, i.e., an increase from 600 to 700 °C and then a decrease for activation at 800 °C. CA-4600 and C-4600 carbons activated at 600 °C have very similar porosity, i.e., apparent surface area of ca. 2000 m^2^ g^−1^, micropore surface area of ca. 1800 m^2^ g^−1^ while pore volume and micropore volume are almost identical at 0.95 and 0.8 cm^3^ g^−1^, respectively. The nitrogen sorption isotherms of the two 600 °C samples are very similar (Supplementary Fig. 4a). The pore size distribution is also similar (Supplementary Fig. 4b), except that the cellulose acetate-derived CA-4600 sample has an ‘extra’ pore maximum centred at 6 Å, which is not present to the same extent for the cellulose-derived C-4600 sample. For activation at 700 °C (Supplementary Fig. 5), the cellulose acetate-derived CA-4700 carbon has much higher surface area (3771 m^2^ g^−1^) and pore volume (1.75 cm^3^ g^−1^) compared to the cellulose-derived C-4700 sample (2370 m^2^ g^−1^ and 1.08 cm^3^ g^−1^, respectively). Thus the surface area and pore volume of the cellulose acetate-derived CA-4700 sample are 60% higher than for the equivalent carbon from cellulose. However, despite this great difference in the overall porosity, the level of microporosity is similar for the two samples at 92% of surface area and 88% of pore volume. The shape of isotherm is similar for the two samples (Supplementary Fig. 5a) and their pore size distribution is comparable (Supplementary Fig. 5b), except for an extra pore maximum centred at 7 Å for the cellulose acetate-derived CA-4700 sample. A similar trend is observed for activation at 800 °C, with the cellulose acetate-derived carbon having higher porosity and the presence of ultramicropores at 6 Å compared to the cellulose-derived analogue (Supplementary Fig. 6).

The extremely high apparent surface area and micropore surface area of sample CA-4700 appears to be unique amongst high surface area porous carbons^24,55,57,61,62^. We, therefore, compared the porosity of sample CA-4700 to that of analogous activated carbons derived from hydrochars of other biomass precursors, namely, glucose^5,7^, starch^5,7^, eucalyptus sawdust^5,7^, furfural^5^, lignin^56^, jujun grass and *Camellia Japonica* ^50^, and also activated carbon derived from carbon nanotube superstructures^57^, all prepared under similar activation conditions, i.e., at 700 °C and KOH/carbon ratio of 4 (Supplementary Fig. 7 and Supplementary Table 3). In all cases, the cellulose acetate-derived CA-4700 carbon exhibits much higher surface area and pore volume than all these other carbons. Thus in addition to generating oxygen-rich carbons, cellulose acetate as a precursor also offers much higher overall porosity whilst retaining a high level of microporosity and in particular small ultramicropores of size 6–7 Å.

### Oxygen functional groups

**Fig. 3:**
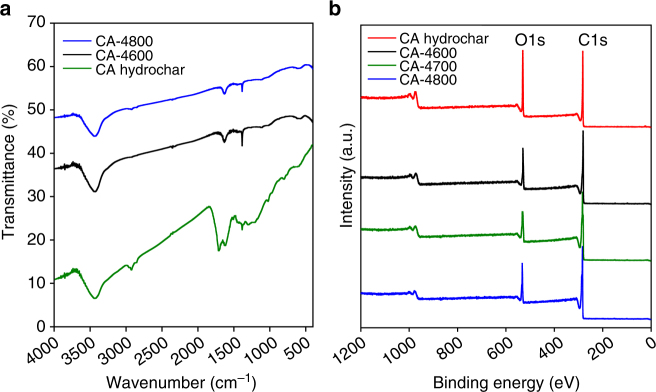
Infra-red and X-ray photoelectron spectroscopy spectra of CA-hydrochar and activated carbons. **a** IR spectra and **b** XPS wide scan spectra of cellulose acetate-derived CA-hydrochar and activated carbons derived from the CA-hydrochar. The activated carbons are labelled as CA-4T, where 4 is the activation KOH/carbon ratio and *T* is the activation temperature in °C

The elemental composition discussed above indicates the presence of high oxygen content in the CA-4T carbons in a manner that is atypical of biomass-derived activated carbons. We used IR spectroscopy to probe the nature of oxygen functional groups on the CA-4T carbons. The IR spectra of the CA-hydrochar and the activated carbons are shown in Fig. 3a. The spectra of both the CA-hydrochar and activated carbons show the C–OH stretch band at ca. 3430 cm^−1^, and C–OH bend band at ca. 1630 cm^−1^ ^7,48,49,64,65^. The spectra of the activated CA-4T carbons also exhibit other peaks attributable to C–O vibrations at 1380 cm^−1^ (sharp peak) and at 1115 and 1025 cm^−1^ ^7,48,49,64,65^. The spectra are consistent with the fact that the activated carbons are oxygen-rich with low amounts of H. When compared to the IR spectra of the CA-hydrochar, the CA-4T samples do not exhibit C–H peaks at ca. 2850 and 1450 cm^−1^, which is consistent with their lower H content (Table 1). We also note that the spectra of the CA-4T samples do not show a clear C=O peak at 1710 cm^−1^, which is present both for the CA-hydrochar (Fig. 3a) and activated carbons derived from sawdust (Supplementary Fig. 8)^55^. The IR spectra confirms the presence of oxygen functional groups, which is consistent with the high oxygen content of the carbons. The IR spectra also show that the CA-4T carbons do not possess any adventitious organic molecules.

To further probe the nature of the oxygen in the CA-4T carbons we performed X-ray photoelectron spectroscopy (XPS). XPS is able to detect which elements are present on the surface of the carbons and how they are bonded to each other. Wide scans were used to quantify the surface elements detected, and only oxygen and carbon were present in detectable quantities as shown in Fig. 3b. The spectra (Fig. 3b) show that both the CA-hydrochar and CA-4T activated carbons contain significant amounts of oxygen on their surface^36,48,65–67^, but that the O-content is lower in the activated carbons. The surface oxygen contents (wt%) estimated from XPS are 20.7% (CA-hydrochar), 14.3% (CA-4600), 13.8% (CA-4700) and 14.8% (CA-4800). These values are lower than the O-content obtained via CHN analysis and may suggest that the surface of the CA-hydrochar and activated carbons are somewhat depleted of oxygen when compared to the bulk. However, it should be noted that most of the surface of the CA-4T carbons is internal (i.e., within the pore channels), and therefore not directly probed by XPS, which may explain the difference in oxygen content when compared to CHN analysis data.

**Fig. 4:**
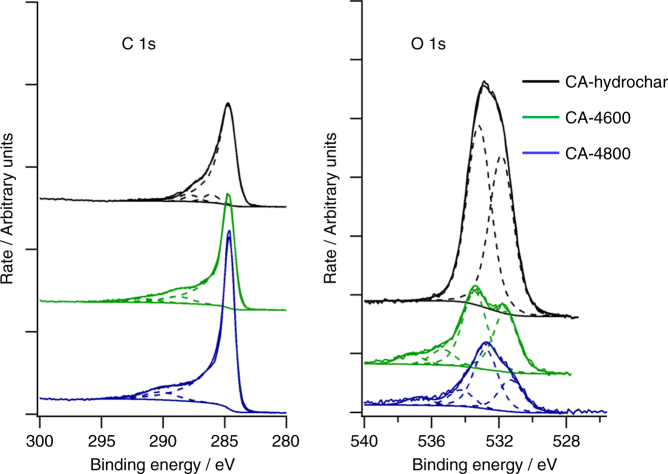
C 1s and O 1s XPS spectra with peak fitting for cellulose acetate-derived CA-hydrochar and activated carbons derived from the CA-hydrochar. The activated carbons are labelled as CA-4T, where 4 is the activation KOH/carbon ratio and *T* is the activation temperature in °C

To obtain higher-resolution XPS spectra, the survey scans were charge corrected to C 1s at 284.5 eV and peak fitted. With the high-resolution spectra it is possible to estimate how much carbon is bonded to oxygen. Figure 4 shows the peak models used for C 1s and O 1s to identify the extent of carbon bonding to oxygen. The atomic % estimated from the high-resolution spectra with a peak fit to define relative amounts of C bonded to oxygen yield ratios of O:C bonded to oxygen of close to 3:1 for the CA-hydrochar, and 1:1 for the activated CA-4600 and CA-4800 carbons. As shown in Fig. 4, the O 1s spectra of the CA-hydrochar is dominated by two components at 531.8 and 533.2 eV. These components have previously been considered as consistent with O–C=O, which may explain the >1 ratio of oxygen bonded to C for the CA-hydrochar^36,48,65–68^. On the other hand, for the CA-4T carbons, the O 1s peaks appear to consist of four components that suggest the presence of other C–O bonding such as COOH and C–OH, which is consistent with assignment in previous reports^36,48,65–68^. The four components, and likely bonding, are consistent with a 1:1 ratio of oxygen bonded to C. The C 1s peak fits are consistent with the presence of C–OH/O–C=O groups on the surface of both the hydrochar and activated carbons^36,48,65–68^. The XPS spectra, is therefore qualitatively in agreement with the elemental (CHN) analysis data and IR spectra regarding the oxygen-rich nature of the CA-4T carbons and the presence of oxygen functional groups.

**Fig. 5:**
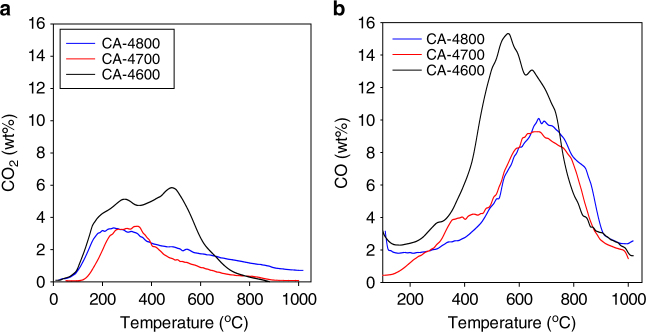
Temperature-programmed desorption of activated carbons. Evolution of **a** CO_2_ and **b** CO under temperature-programmed desorption (TPD) conditions for activated carbons derived from cellulose acetate. The activated carbons are labelled as CA-4T, where 4 is the activation KOH/carbon ratio and *T* is the activation temperature in °C

To further probe the nature of the carbons and their oxygen content, we performed temperature programmed desorption (TPD). The TPD profiles for CO_2_ and CO are shown in Fig. 5. The CO_2_ is desorbed in the temperature range 150–550 °C, while the CO is desorbed at between 400 and 900 °C. The observed TPD profiles are similar to what has been previously observed for activated carbons^46^. It is expected that the desorbed CO_2_ is from less thermally stable O-containing functional groups such as carboxylic acids, lactones and anhydrides. On the other hand, the CO is evolved from carbonyls, quinonic or phenolic groups^46^. The oxygen content estimated from the TPD data is very similar to that from elemental analysis (Table 1), being 22.3 for CA-4600, 18.5 for CA-4700 and 21.2 for CA-4800. The TPD data are thus consistent with the XPS, FTIR and elemental analysis as discussed above and are further evidence of the oxygen-rich nature of the present carbons.

### Hydrogen storage

Hydrogen storage in porous carbon requires materials with the highest surface area and appropriately sized pores. To date, the best performing porous carbons include zeolite-templated carbons^9,10,22,30,33^, carbide-derived carbons (CDCs)^69^ and activated carbons^5,7,21,23,55,56^. The hydrogen storage capacity of the CA-4T-activated carbons was investigated at −196 and 25 °C using a gravimetric method on a Hiden XEMIS analyser in the pressure range of 0–30 bar. Our assessment conditions (−196 or 25 °C, and 0–30 bar) were informed by the fact that cryo-storage has recently gained strong consideration as being viable for low-pressure vehicular hydrogen storage^70,71^, and also the fact that near-ambient temperature storage is the most preferred option^1–3,72,73^. The values discussed in this report are material-based on a dry material basis.

**Fig. 6:**
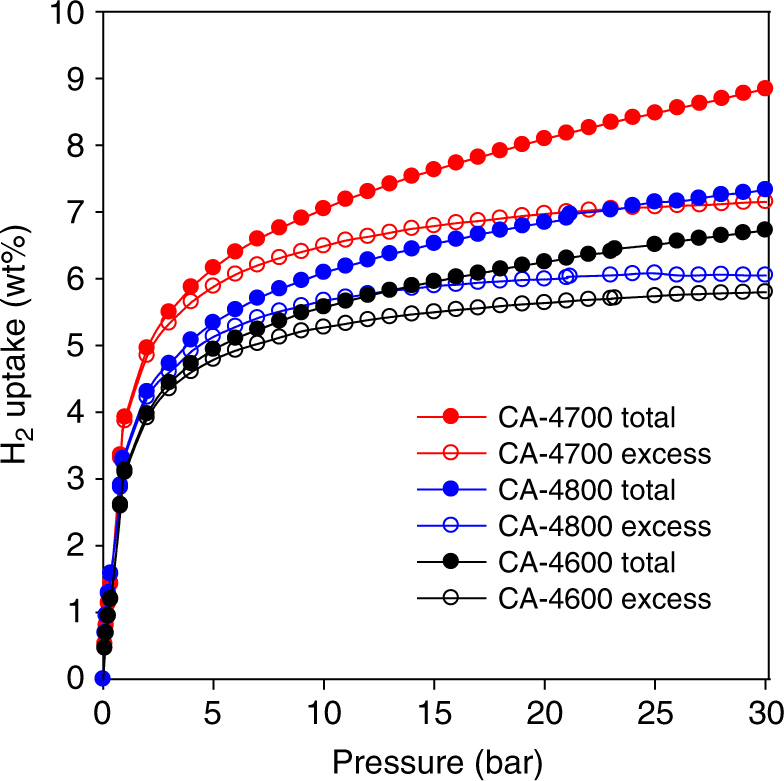
Gravimetric hydrogen storage capacity of activated carbons. Excess and total hydrogen uptake at −196 °C of activated carbons derived from cellulose acetate

Our gravimetric method measured the excess hydrogen uptake from which the total storage was obtained (see Methods section for details on how excess and total hydrogen uptake were obtained). Figure 6 shows the excess and total hydrogen uptake isotherms at −196 °C, and the uptake at 1, 20 and 30 bar is summarised in Table 2. For all the CA-4T carbons, the hydrogen uptake is reversible, with no hysteresis, and no saturation is attained in the 0–30 bar pressure range. We first note that the hydrogen uptake at 1 bar is very impressive, being in the range 3.1–3.9 wt%. Such hydrogen storage is much higher than any reported values for high surface area porous carbons that tend to be in the range of 2–3 wt%^5–9,23,55^. We attribute this exceptionally high uptake, especially for sample CA-4700 at 3.9 wt%, to a combination of very high micropore surface area and an oxygen-rich surface. The effect of an oxygen-rich surface is particularly apparent for sample CA-4600, which despite having a moderate total surface area and micropore surface, still exhibits uptake that is much higher than that of previously reported carbons with much higher surface area^5–9,23,55^. A positive effect of having oxygen functional groups, as observed here, is expected to have the greatest effect at low pressure, where interaction between the hydrogen and surface is more important than at higher pressure where hydrogen uptake is more likely to occur via space filling mechanisms. At 20 bar, sample CA-4600 has an excess hydrogen uptake of 5.6 wt%, which rises to 7.0 wt% for CA-4700 and then reduces to 6.0 wt% for CA-4800. These excess hydrogen uptake values are the highest ever reported for porous carbons; the best previous reports have been in the range 5–5.5 wt%^5–9,23,55^. Sample CA-4700 stands out with excess uptake of 7.0 wt% (−196 °C and 20 bar), which is ~30% higher than any previously reported carbon^5–9,23,55^. We attribute the high excess uptake to a combination of very high micropore surface area and an oxygen-rich surface. The total hydrogen uptake at 20 bar is 6.2 wt% for sample CA-4600, 8.1 wt% for CA-4700 and 6.8 wt% for CA-4800. A total hydrogen uptake of 8.1 wt% at 20 bar for sample CA-4700 sets a new record for porous carbons. The highest reported values to date are 7.08 wt% for a doubly activated carbon (physical activation followed by activation with KOH)^23^, 7.03 wt%^29^, and 7.3 wt%^55^, respectively, for activated and compactivated carbons derived from polypyrrole, 7.1 wt% for a compactivated carbon derived from sawdust^55^, and 7.3 wt% for a zeolite-templated carbon^9^. At 30 bar, excess hydrogen uptake of the samples rises to 5.8 wt% (CA-4600), 7.2 wt% (CA-4700) and 6.1 wt% (CA-4800). The corresponding total hydrogen storage values are very impressive at 6.7 wt% (CA-4600), 8.9 wt% (CA-4700) and 7.3 wt% (CA-4800). The uptake of sample CA-4700 stands out as a new record for carbons under such measurement conditions^55^.

In all cases, the hydrogen uptake of the cellulose acetate-derived CA-4T carbons is much higher than that of the analogous cellulose-derived C-4T-activated carbons (Supplementary Fig. 9; Supplementary Table 2). The comparison between CA-4600 and C-4600 is interesting because, although the two samples have similar apparent surface area and pore volume (Supplementary Table 2), the former has much higher hydrogen uptake; at 1 bar the uptake of CA-4600 (3.1 wt%) is 35% higher than for C-4600 (2.3 wt%) while at 20 bar it is higher by ca. 30% for both excess and total storage. The main difference in the porosity of the two samples is that the cellulose acetate-derived C4-4600 sample has a pore maximum centred at 6 Å, which is not present to the same extent for the cellulose-derived C-4600 sample (Supplementary Fig. 4). We have previously shown that carbons that possess a significant proportion of ultramicropores of size 6 Å exhibit higher hydrogen uptake^10^. We also partly ascribe the higher uptake of sample CA-4600 to having an oxygen-rich surface, which is consistent with previous reports on related systems^36–40^. At 1 bar the hydrogen uptake of CA-4700 (3.9 wt%) and CA-4800 (3.4 wt%) are up to 60% higher than for the equivalent C-4700 (2.5 wt%) and C-4800 (2.1 wt%) samples, while at 20 bar, the storage capacity (both excess and total) of the CA-4T carbons is ca. 45% higher (Supplementary Table 2). We attribute this higher hydrogen uptake to a combination of overall higher porosity, the presence of ultramicropores (Supplementary Figs. 5 and 6) and oxygen-rich nature for the CA-4T samples. More generally, sample CA-4700 has much higher hydrogen uptake compared to analogous activated carbons derived from a wide range of precursors (Supplementary Table 3) and prepared under similar activation conditions (i.e., at 700 °C and KOH/carbon ratio of 4).

To further clarify on the effect of oxygen functional groups and ultramicropores, we compared (Supplementary Fig. 10; Supplementary Table 4) the hydrogen uptake of sample CA-4700 to that of benchmark high surface area carbons with low (PPY-4800)^55^ or high (ZTC5)^9^ microporosity, and a benchmark commercially available activated carbon (AX21) that exhibits high surface area (Supplementary Fig. 11; Supplementary Table 4). The hydrogen uptake of sample CA-4700 is clearly higher than for the benchmark carbon materials (Supplementary Table 4). Despite a high surface area, sample PPY-4800 is mainly mesoporous meaning it has no ultramicropores and therefore has a relatively low hydrogen uptake at 1 bar (2.2 wt%) and moderate excess hydrogen uptake (5.5 wt%) at 20 bar compared to 3.9 and 7.0 wt%, respectively, for CA-4700. Sample AX21 also has high surface area and pore volume with ca. 50% of the porosity arising from micropores, but exhibits comparatively low hydrogen uptake at 1 bar (2.1 wt%) and modest excess hydrogen uptake (4.7 wt%) at 20 bar. On the other hand sample ZTC5 is highly microporous but still does not reach the hydrogen uptake of CA-4700; here the main cause of the difference in hydrogen uptake is likely to be the oxygen-rich nature of the CA-4700 sample. Assessment of the hydrogen uptake of the commercially available activated carbon (AX21) also serves to validate our storage data collection method. The hydrogen uptake measured by the XEMIS for sample AX21 at 20 bar is 4.7 wt% (excess uptake) and 5.9 wt% (total uptake), which is similar to that reported by others for this commercially available activated carbon using volumetric apparatus^12^. Furthermore, we have recently demonstrated the reliability of our hydrogen uptake measurements via comparison with deuterium uptake^55^. Validation and benchmarking of hydrogen storage data is important given the challenges that have been encountered in the recent past research as summarised by Broom and Hirscher^74^.

Given that the CA-4T samples are both highly microporous and oxygen-rich, it is necessary to delineate the effect of microporosity and oxygen content on the hydrogen uptake capacity. One way to ascertain the effects of oxygen content is to establish how the hydrogen uptake capacity of CA-4T carbons compares to that of activated carbons with lower amounts of oxygen but similar porosity (i.e., similar total surface area, micropore surface area, pore size and level of microporosity). Such a comparison will remove any ambiquities and clarify on the effect of oxygen content. We therefore compared the hydrogen uptake of sample CA-4800 to analogous activated carbons derived from the hydrochar of Jujun grass (ACGR4700) and *Camellia Japonica* (ACCA4700)^50^ that have closely matched porosity but lower oxygen content (Supplementary Fig. 12; Supplementary Table 5). The total surface area of the three samples is very similar and in the range 3000 ± 5% m^2^ g^−1^, while the micropore surface area is within 2620 ± 5% m^2^ g^−1^ (Supplementary Table 5). Such variability (i.e., ±5%) in total surface area and micropore surface area is within the repeatability range of the method used to determine porosity, and therefore the surface area of the three samples is comparable. The total pore volume of the three samples is also closely matched at 1.44 ± 5% cm^3^ g^−1^ and, more importantly, the micropore volume is even more closely matched at 1.18 ± 4% cm^3^ g^−1^ (Supplementary Table 5). Furthermore, the pore size of all three samples is also very similar (Supplementary Fig. 12). The main difference is that sample CA-4800 has a much higher O-content of 20.6 wt% compared to 13.3 wt% for ACGR4700 and 12.5 wt% for ACCA4700 (Supplementary Table 5), i.e., the oxygen content of CA-4800 is ca. 60% higher than for the other two samples. Despite the similarity in porosity, sample CA-4800 exhibits significantly higher hydrogen uptake at −196 °C, while ACGR4700 and ACCA4700 have lower but identical storage capacity (Supplementary Table 5). At 1 bar CA-4800 stores 3.4 wt% compared to 2.4 wt% for ACGR4700 and ACCA4700, which is a difference of 40% for the CA-4T sample. At 20 bar the excess uptake of CA-4800 is 6.0 wt% compared to 4.5 wt% for ACGR4700 and ACCA4700, meaning the CA-4T sample has 30% higher capacity.

Ideal hydrogen storage materials for vehicular on-board applications need to achieve targets set at levels that would allow commercial viability and practical usage^75^. Targets include those set by the United States Department of Energy (DOE); namely, a system uptake capacity of 5.5 wt% and volumetric uptake capacity of 40 g l^−1^ by 2020, and ultimate targets of 7.5 wt% (system gravimetric storage capacity) and 70 g l^−1^ (volumetric uptake capacity)^75^. Whilst the gravimetric hydrogen uptake of porous materials generally depends on the porosity, especially surface area and pore size, the volumetric hydrogen uptake, on the other hand, is greatly affected by the packing density of the adsorbent. The packing density of the CA-4T samples was determined (see Methods) to be 0.71 g cm^−3^ (CA-4600), 0.46 g cm^−3^ (CA-4700) and 0.56 g cm^−3^ (CA-4800). The volumetric hydrogen uptake of the CA-4T carbons is given in Table 2. All the samples show excellent volumetric hydrogen storage capacity at 20 bar; 32–40 g l^−1^ (excess) and 37–44 g l^−1^ (total). At 30 bar the uptake is 33–41 g l^−1^ (excess) and 41–48 g l^−1^ (total). We, however, note that although these values are impressive, they are material-based and would need to be higher to meet the system-based DOE targets.

It is interesting to note that the gravimetric hydrogen uptake of the CA-4T carbons, and in particular sample CA-4700, compares favourably with that of the best performing MOFs that possess much higher surface area (Supplementary Fig. 13; Supplementary Table 6). The excess (7.0 wt%) and total (8.1 wt%) gravimetric hydrogen uptake of sample CA-4700 at 20 bar is higher than that of benchmark MOFs such as MOF-200^16^, MOF-205^16^, and comparable to that of NOTT-112 (6.9 wt%)^13^, NU-100 (6.8 wt%)^17^ and MOF-210 (6.4 wt%)^16^, which have much higher surface area (Supplementary Table 6) and are the current record holders for gravimetric hydrogen storage in porous materials under cryogenic conditions^16^. The total gravimetric uptake at 20 bar follows a similar trend. We tentatively ascribe the excellent hydrogen storage capacity of sample CA-4700, despite having lower surface area, to an oxygen-rich surface that appears to be more attractive for adsorption of molecular hydrogen, and which more than compensates for the differences in porosity compared to the MOFs. At 30 bar, the excess gravimetric hydrogen uptake of CA-4700 still matches that of the high surface area MOFs (Supplementary Table 6) but the total uptake of some of the MOFs is higher due to greater contribution of their much larger pore volume.

**Fig. 7:**
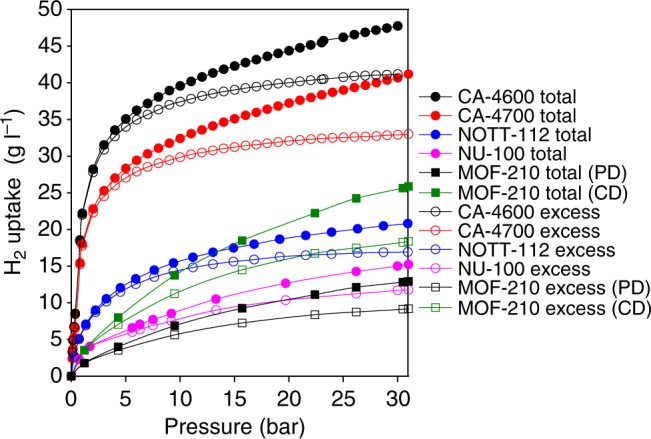
Volumetric hydrogen storage capacity of activated carbons compared to benchmark metal organic framework materials. Excess and total volumetric hydrogen uptake of activated carbons (CA-4600 and CA-4700) derived from cellulose acetate compared to benchmark high surface area metal organic frameworks (MOFs), namely, NOTT-112^13^, NU-100^17^ and MOF-210^16^. PD means packing density, and CD means crystal density was used for calculation of volumetric uptake

More importantly, however, the higher packing density of the CA-4T carbons, i.e., 0.71 g cm^−3^ (CA-4600) 0.46 g cm^−3^ (CA-4700) and 0.56 g cm^−3^ (CA-4800) when combined with their impressive gravimetric uptake, means that their volumetric hydrogen storage capacity is much higher than that of MOFs. As shown in Fig. 7 (and Supplementary Table 6), the benchmark MOFs achieve excess volumetric hydrogen uptake of 8–16 g l^−1^ at 20 bar, and 9–17 g l^−1^ at 30 bar. In contrast, the excess volumetric capacity of the CA-4T carbons is much higher at 32–40 g l^−1^ (20 bar), and 33–41 g l^−1^ at 30 bar. The total volumetric hydrogen uptake of the MOFs is 11–19 g l^−1^ at 20 bar, and 13–21 g l^−1^ at 30 bar compared to 37–44 g l^−1^ at 20 bar, and 41–48 g l^−1^ at 30 bar for the CA-4T carbons. In Fig. 7 (and Supplementary Table 6), we also applied the crystal density to calculate the volumetric uptake of MOF-210– but at up to 22 and 26 g l^−1^ for excess and total uptake at 30 bar, it is still lower than that of the CA-4T carbons. Thus the volumetric uptake of the CA-4T carbons under our analysis conditions is double that of the best MOFs, and is amongst the highest reported. The only other carbons that come close are compactivated carbons^55^, densified zeolite templated carbons^9^, and densified activated ZIF-templated carbons^27^. The CA-4T carbons are, therefore, a significant step towards achieving the DOE targets for 2020 of a system gravimetric uptake capacity of 5.5 wt% and volumetric uptake capacity of 40 g l^−1^ ^75^. Clearly, higher storage values that may approach the ultimate targets of a 7.5 wt% (system gravimetric storage capacity) and 70 g l^−1^ (volumetric uptake capacity) may be reached by CA-4T carbons at higher pressures (>30 bar)^75^.

**Fig. 8:**
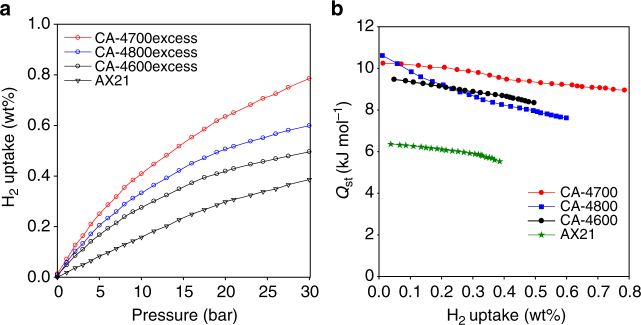
Room temperature hydrogen storage capacity and isosteric heat of hydrogen adsorption. **a** Gravimetric excess hydrogen uptake at 25 °C of the CA-4T-activated carbons derived from cellulose acetate and a commercially available activated carbon AX21, and **b** isosteric heat of hydrogen adsorption (*Q*_st_) as a function of hydrogen uptake for the CA-4T activated carbons derived from cellulose acetate and a commercially available activated carbon AX21

Any beneficial effects of oxygen surface groups on hydrogen storage might be expected to have a significant effect on room temperature uptake. The room temperature excess hydrogen uptake of the CA-4T samples and AX21 are shown in Fig. 8a. The excess hydrogen uptake, at 20 bar, varies from 0.3 wt% for AX21 to 0.42 wt% (CA-4600), 0.5 wt% (CA-4-800) and 0.63 wt% (CA-4700). We note that the excess uptake for AX21 (0.3 wt% at 20 bar) is similar to what has previously been reported by others using volumetric apparatus, which verifies our measurements^12^. At 30 bar, the excess uptake rises to 0.38 wt% for AX21, 0.50 wt% (CA-4600), 0.61 wt% (CA-4-800) and 0.80 wt% (CA-4700). The CA-4T samples, therefore have higher uptake compared to what has previously been reported for porous carbons or related materials^12,55,57,76–78^. The excess hydrogen storage capacity of 0.8 wt% at room temperature and 30 bar for sample CA-4700 is exceptionally high for any porous material under those conditions and even gets close to what has previously been reported (between 0.5 and 1.6 wt%) for the best performing state-of-the-art materials at much higher pressure of 150 bar^12,55,57,76–78^. We also estimated the total uptake of the CA-4T carbons and sample AX21 (Supplementary Fig. 14). The total uptake, at 30 bar, reaches 0.8 wt% for AX21, but is higher for CA-4800 and CA-4700 at 0.95 wt% and 1.2 wt%, respectively. It is expected that CA-4T samples, for *T* = 700 and 800 °C, will have attractive excess and total hydrogen uptake at high pressure above 30 bar as shown by the example of sample CA-4700 (Supplementary Fig. 15).

The strength of the interaction between adsorbed hydrogen molecules and the carbon surface can be estimated by calculating the so-called isosteric heat of hydrogen adsorption (*Q*_st_). The *Q*_st_ may be calculated from the hydrogen sorption isotherms measured at two or more temperatures, and the typical temperatures that are used are −196, −186 and 25 °C. We, therefore, calculated the *Q*_st_ for the CA-4T samples using the Clausius–Clapeyron equation. A plot of the *Q*_st_ as a function of hydrogen uptake is shown in Fig. 8b. We first note that the *Q*_st_ for the commercially available activated carbon (AX21) varies from 6.4 kJ mol^−1^ at low (i.e., near zero) hydrogen uptake and then decreases to 5.5 kJ mol^−1^ at coverage equivalent to ca. 0.4 wt% hydrogen. The *Q*_st_ for AX21 is very similar to that previously reported by others^79^, which is very useful in benchmarking the CA-4T carbons. The *Q*_st_ for the CA-4T samples is significantly higher than that of AX21. Amongst the CA-4T sample, CA-4600 has the lowest *Q*_st_, which ranges from 9.5 kJ mol^−1^ at low coverage to 8.3 kJ mol^−1^ at 0.5 wt%. For CA-4700 the *Q*_st_ is 10.2 kJ mol^−1^ at low coverage, decreasing to 9.0 kJ mol^−1^ at 0.8 wt% hydrogen. Sample CA-4800 exhibits the highest *Q*_st_ of 10.6 kJ mol^−1^ at near zero coverage, but also shows the most significant decrease as coverage increases to 7.6 kJ mol^−1^ at 0.6 wt% hydrogen. As far as we know the *Q*_st_ values reported here for the CA-4T samples are significantly higher than in any previous reports concerning porous carbons, and in particular activated carbons. The *Q*_st_ are only matched by those reported by Gogotsi and co-workers for some carbide-derived carbons^69^. We ascribe the high *Q*_st_ of the CA-4T carbons to not only their microporosity but also their oxygen-rich nature. Regarding the effect of microporosity, we note that the CA-4T carbons have higher *Q*_st_ than zeolite-templated carbons with a similar level of microporosity that only reach presence ca. 8.2 kJ mol^−1^ at near zero coverage^8^. This, along with the optimised hydrogen uptake at room temperature, provide further evidence of the effect of an oxygen-rich surface on the hydrogen uptake.

## Discussion

Our findings show that hydrothermal carbonisation of cellulose acetate generates hydrochar, which on activation yields oxygen-rich activated carbons that exhibit very high apparent surface area (up to 3800 m^2^ g^−1^) with most of the surface area (up to 3484 m^2^ g^−1^), i.e., 92%, arising from micropores. The carbons have only a small proportion of pores of size larger than 20 Å. The presence of oxygen functional groups (COOH, C–OH and O–C=O) was confirmed by IR and XPS studies. Comparative analysis with carbons from other precursors (e.g., cellulose, lignin, sawdust, starch and so on) revealed that the oxygen-rich nature and high microporosity is unique to cellulose acetate-derived activated carbons. Due to the combined effects of high surface area, high microporosity and an oxygen-rich nature, the carbons exhibit enhanced gravimetric hydrogen storage capacity of up to 8.1 wt% (total uptake) and 7.0 wt% (excess uptake) at −196 °C and 20 bar, rising to 8.9 wt% (total uptake) and 7.2 wt% (excess uptake) at 30 bar. The carbons also exhibit exceptional hydrogen uptake at room temperature; up to 0.8 wt% (excess) and 1.2 wt% (total) at only 30 bar. The isosteric heat of hydrogen adsorption for the carbons is above 10 kJ mol^−1^, which is nearly twice that typically observed for activated carbons. The hydrogen uptake values set new records for porous carbon materials and exceed the claimed limit for hydrogen storage in activated carbons^80^. The high gravimetric hydrogen uptake values at −196 °C, translate to attractive total volumetric uptake of 37–44 g l^−1^ at 20 bar, and 41–48 g l^−1^ at 30 bar compared to benchmark metal organic frameworks (MOFs) that store 11–19 g l^−1^ at 20 bar, and 13–21 g l^−1^ at 30 bar. Our findings offer new insights on the effect of precursor materials on activated carbons prepared via the hydrothermal carbonisation route, and on the benefits of an oxygen-rich nature on hydrogen storage in porous carbons, and open up a new research direction in carbon materials as hydrogen stores.

## Methods

### Materials synthesis

Hydrochar suitable for activation was prepared by heating 6.4 g of cellulose acetate in 20 ml of water in a stainless steel autoclave to 250 °C at a ramp rate of 5 °C min^−1^. The temperature was held at 250 °C for 2 h and then brought back to room temperature at a ramp rate of 5 °C min^−1^. The hydrochar product was then filtered under suction and washed with water. The hydrochar, denoted as CA-hydrochar, was dried overnight in an oven at 112 °C.

The hydrochar was then activated as follows; KOH was added to the hydrochar at a KOH/hydrochar ratio of 4 and ground to form a homogenous mixture. The KOH/hydrochar mixture was then placed in an alumina boat and transferred to a horizontal furnace and heated under nitrogen to 600, 700 or 800 °C at a ramp rate of 3 °C min^−1^, and held at the target temperature for 2 h before being allowed to cool under nitrogen. The resulting activated carbons were washed under stirring at room temperature with hydrochloric acid (10 vol%, 500 ml) and then with deionised water until washings had neutral pH. All samples were then dried at 112 °C. The activated carbons were denoted as CA-4T, where 4 is the KOH/hydrochar ratio and T is the activation temperature; 600, 700 or 800 °C.

### Materials characterisation

Powder XRD analysis was performed using a PANalytical X’Pert PRO diffractometer with Cu-Kα light source (40 kV, 40 mA) with step size of 0.02° and 50 s time step. Thermogravimetric analysis (TGA) was performed using a TA Instruments SDT Q600 analyser under flowing air conditions (100 ml/min). CHN elemental analysis was performed using an Exeter Analytical CE-440 Elemental Analyser. IR spectroscopy was performed using a Bruker ALPHA FTIR Spectrometer. Raman spectra were recorded using a Horiba–Jobin–Yvon LabRAM Raman microscope with a 532 nm laser operating at ca. 4 mW (10%) and a 600 lines per mm grating. Spectra were collected by averaging 8 acquisitions of 60 s duration. The Raman shift was calibrated using the Rayleigh peak and the 520.7 cm^−1^ Si line from a Si (100) reference sample. X-ray photoelectron spectroscopy (XPS) was performed using a Kratos AXIS ULTRA with a mono-chromated Al k*α* X-ray source (1486.6 eV) operated at 10 mA emission current and 12 kV anode potential. A charge neutralizer filament was used to prevent surface charging. Hybrid-slot mode was used measuring a sample area of approximately 0.5 mm^2^. The analysis chamber pressure was better than 5 × 10^−9^ mbar. Three areas per sample were analysed. A wide scan at low resolution (1400 to −5 eV binding energy range, pass energy 80 eV, step 0.5 eV, sweep time 20 min) was used to estimate the total atomic % of detected elements. High-resolution spectra at pass energy of 20 eV with step of 0.1 eV, sweep times of 10 min each were also acquired for photoelectron peaks from the detected elements and these were used to model the chemical composition. The high-resolution spectra were charge corrected to the C 1s peak set to 284.7 eV. Casaxps (version 2.3.18dev1.0x) software was used for quantification and spectral modelling. For detector mode, the ULTRA was used in FAT (fixed analyser transmission) mode, with pass energy of 80 eV for wide scans and pass energy 20 eV for high-resolution scans. A hybrid magnetic/electrostatic lens mode was used for maximum electron signal. The magnetic immersion lens system allowed the area of analysis for to be defined by apertures, a ‘slot’ aperture of 300 × 700 μm for wide/survey scans and high-resolution scans. The take-off angle for the photoelectron analyser was 90° and acceptance angle of 9° for hybrid lens mode utilised. The energy range of the XPS was calibrated using Cu, Ag and Au samples. The resolution for the photoelectron detector was based on Ag 3d_5/2_ peak FWHM of <0.55 eV at PE 20 in hybrid slot mode. The transmission function of the instrument was calibrated using a clean gold sample for all lens modes and pass energies.

Porosity analysis and determination of textural properties was performed via nitrogen sorption using a Micromeritics ASAP 2020 or 3FLEX sorptometer. Prior to analysis (at −196 °C), the carbon samples were degassed under vacuum at 200 °C for 12 h. The apparent surface area (herein referred to simply as surface area) was calculated using the Brunauer–Emmett–Teller (BET) method applied to adsorption data in the relative pressure (*P/P*_*o*_) range 0.04–0.22. The total pore volume was determined from the nitrogen uptake at close to saturation pressure (*P/P*_*o*_ ≈ 0.99). The micropore surface area and micropore volume were determined via *t-*plot analysis. To assess reproducibility, the synthesis of the carbon samples was repeated at least once; the surface area and pore volume were, in general, within ±5% for repeated synthesis or measurements, which is consistent with the repeatability range (or experimental error) of the method used to determine porosity. Non-local density functional theory (NL-DFT) was applied to nitrogen adsorption isotherms to determine pore size distribution. SEM images were recorded on a FEI Quanta200 microscope at 5 kV accelerating voltage.

### Hydrogen uptake measurements

Hydrogen uptake capacity of the carbons was measured by gravimetric analysis with a Hiden XEMIS Intelligent Gravimetric Analyser using 99.9999% purity hydrogen additionally purified by a molecular sieve filter. Prior to analysis, the carbon samples were dried in an oven for 24 h at 80 °C and then placed in the analysis chamber and degassed at 200 °C and 10^−10^ bar for 4–6 h. The hydrogen uptake measurements were performed at −196 °C (in a liquid nitrogen bath) or room temperature (25 °C) over the pressure range of 0–30 or 100 bar.

Our measurements provided the excess hydrogen uptake, which is the amount of hydrogen adsorbed in the carbons above that which would have been stored in the pores under similar conditions (temperature and pressure) assuming that there is zero energy of interaction between the hydrogen and the carbon pore walls. The total uptake was calculated from the excess storage by taking into account the amount of hydrogen compressed into the carbon pore volume space. Our gravimetric methods measured the excess hydrogen uptake (*θ*_Exc_) from which the total storage (*θ*_T_) was calculated from the equation:

1 $$\theta _{\mathrm{T}} = \theta _{{\mathrm{Exc}}} + \frac{{100 \times d_{{\mathrm{H2}}} \times V_{\mathrm{T}}}}{{1 + d_{{\mathrm{H2}}} \times V_{\mathrm{T}}}}$$

where

*θ*_T_ = total hydrogen uptake (wt%)

*θ*_Exc_ = excess hydrogen uptake (wt%)

*d*_H2_ = density (g cm^−3^) of compressed hydrogen gas at the relevant temperature and pressure. The density was obtained from the National Institute of Standards and Technology (NIST) website (http://www.nist.gov/)

*V*_T_ = pore volume (cm^3^ g^−1^) of the carbon from nitrogen sorption analysis.

The excess volumetric uptake capacity (*vθ*_Exc_), in g l^−1^, was obtained using the equation;

2 $$v\theta _{{\mathrm{Exc}}} = \theta _{{\mathrm{Exc}}} \times d_{{\mathrm{carbon}}}$$

where *d*_carbon_ = packing density of the carbon.

The total volumetric uptake capacity (*vθ*_T_), in g l^−1^, was obtained using the equation;

3 $$v\theta _{\mathrm{T}} = \theta _{\mathrm{T}} \times d_{{\mathrm{carbon}}}$$

where *d*_carbon_ = packing density of the carbon

The packing density of powder samples was determined by pressing a given amount of carbon in a 1.3 cm die at pressure of 7 MPa for 5 min. Alternatively similar values can be obtained using the general equation; *d*_carbon_ = (1/*ρ*_s_ + *V*_T_)^−1^, where *ρ*_s_ is skeletal density and *V*_T_ is total pore volume.

### Data availability

Data that support the findings of this study are available within the paper (and its Supplementary Information Files) and from the corresponding author on reasonable request.

## Electronic supplementary material

Supplementary Information
