# Author Correction: Oxygen-rich microporous carbons with exceptional hydrogen storage capacity

**DOI:** 10.1038/s41467-021-26590-4

**Published:** 2021-10-29

**Authors:** L. Scott Blankenship, Norah Balahmar, Robert Mokaya

**Affiliations:** School of Chemistry, University of Nottingham, University Park, Nottingham, NG7 2RD UK

Correction to: *Nature Communications* 10.1038/s41467-017-01633-x, published online 16 November 2017.

In this article the author name L. Scott Blankenship was incorrectly written as Troy Scott Blankenship II. The original article has been corrected.

