# Supplementary material for: Oxygen-rich microporous carbons with exceptional hydrogen storage capacity
Source: Nat Commun. 2017 Nov 16;8:1545. doi: 10.1038/s41467-017-01633-x (PMC5691040; doi:10.1038/s41467-017-01633-x)
Supplement: Supplementary file 1 — Supplementary Information [file 41467_2017_1633_MOESM1_ESM.pdf]

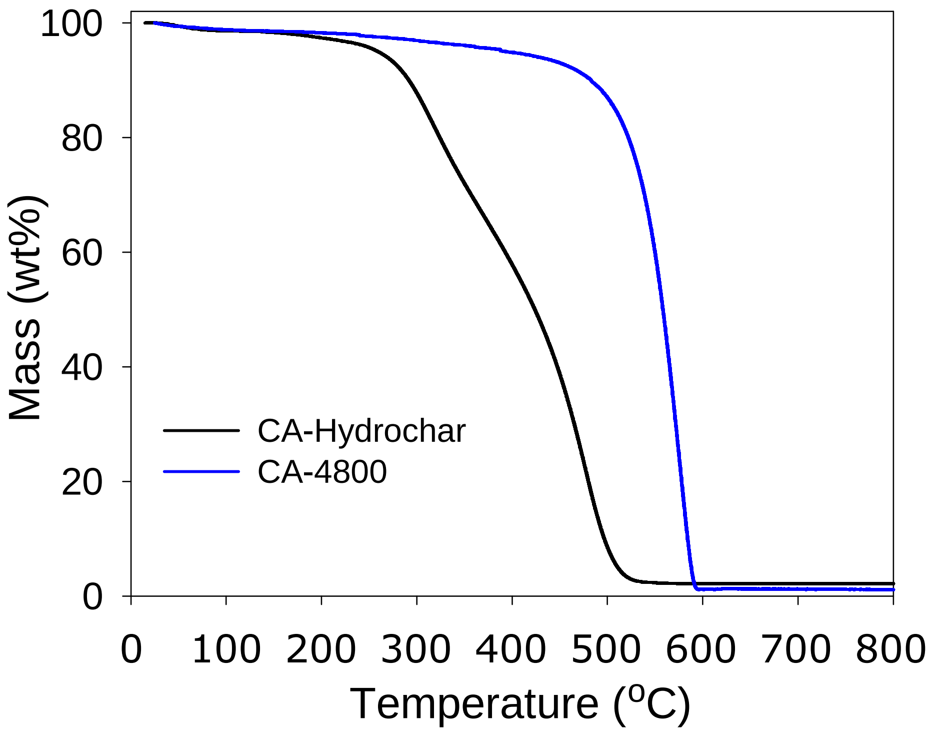

Supplementary Figure 1. Thermogravimetric analysis (TGA) curve of CA-hydrochar prepared from cellulose acetate and activated carbon (CA-4800) derived from the hydrochar via activation at 800 °C and KOH/hydrochar ratio of 4.

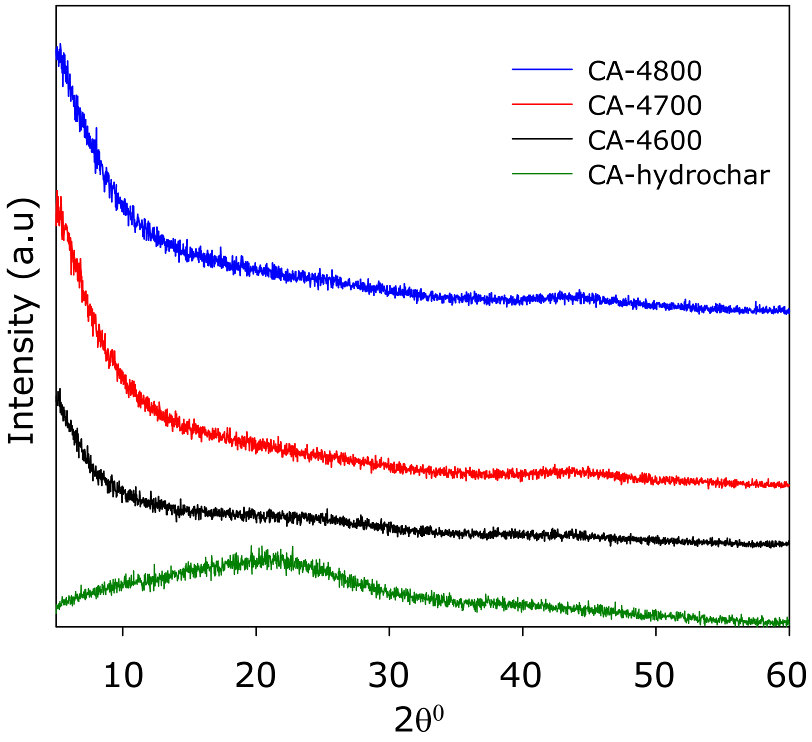

Supplementary Figure 2. Powder XRD patterns of CA-hydrochar and activated carbons prepared from cellulose acetate.

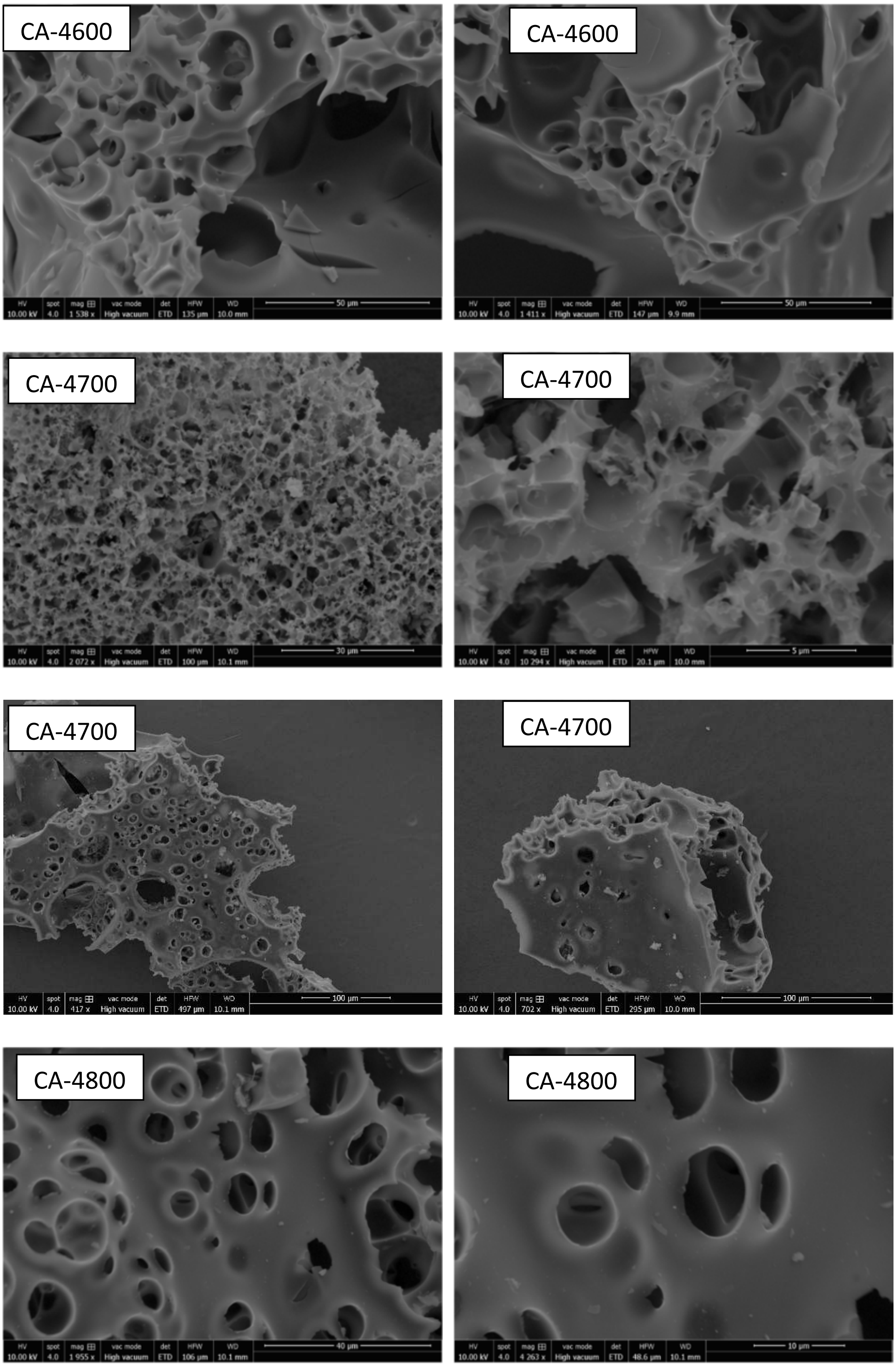

Supplementary Figure 3. SEM images of activated carbons prepared from cellulose acetate. The carbons were prepared from activation at various temperatures (600 – 800 ^oC) of cellulose acetate-derived hydrochar at KOH/hydrochar ratio of 4.

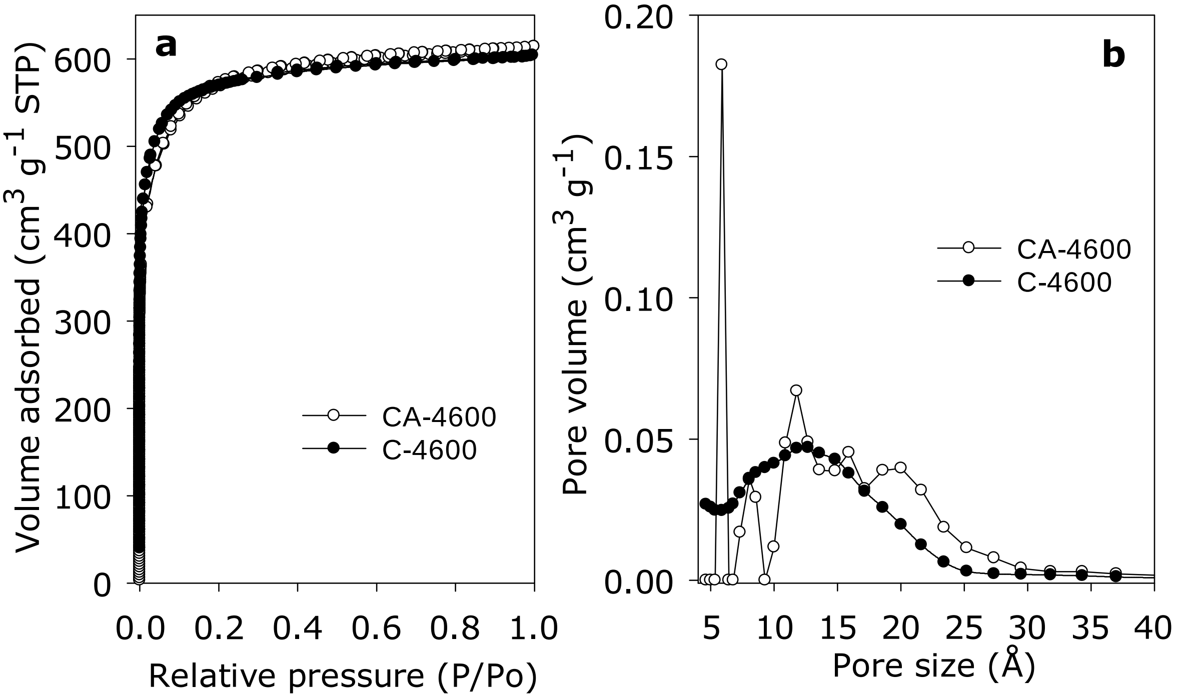

Supplementary Figure 4. Porosity of activated carbons prepared under similar conditions but from different precursors. (a) Nitrogen sorption isotherms and (b) pore size distribution curves of activated carbons prepared from cellulose acetate (CA-4600) or cellulose (C-4600) by activation of their respective hydrochars at 600 °C and KOH/hydrochar ratio of 4.

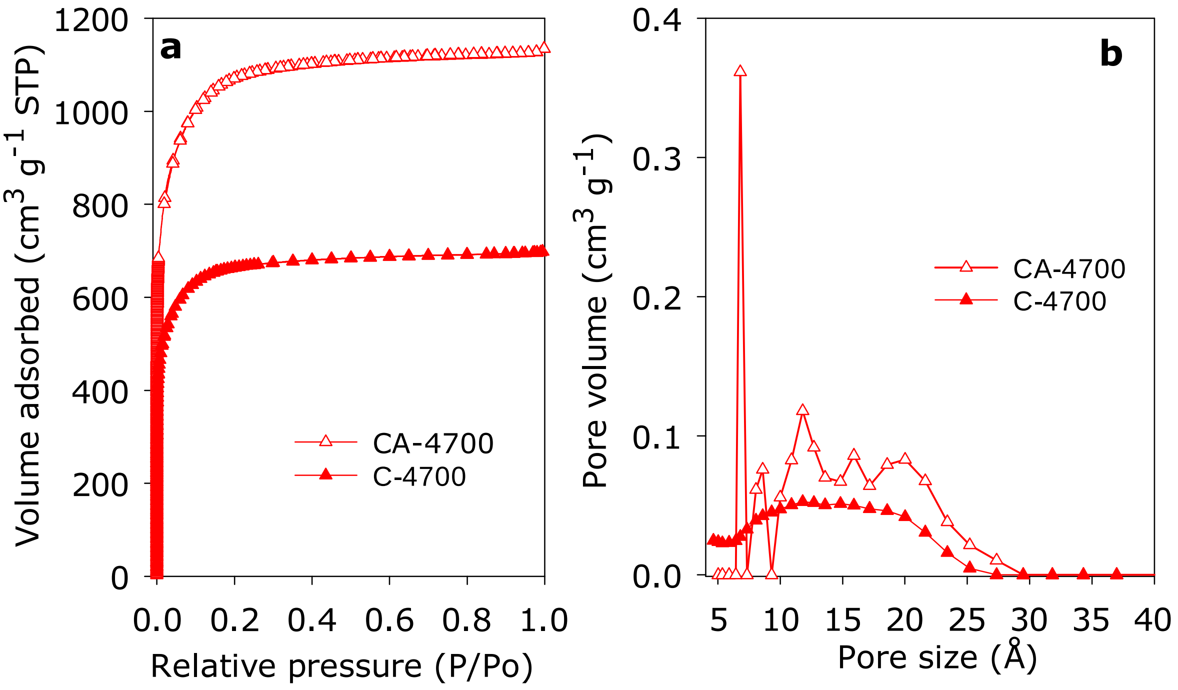

Supplementary Figure 5. Porosity of activated carbons prepared under similar conditions but from different precursors. (a) Nitrogen sorption isotherms and (b) pore size distribution curves of activated carbons prepared from cellulose acetate (CA-4700) or cellulose (C-4700) by activation of their respective hydrochars at 700 ^{o}C and KOH/hydrochar ratio of 4.

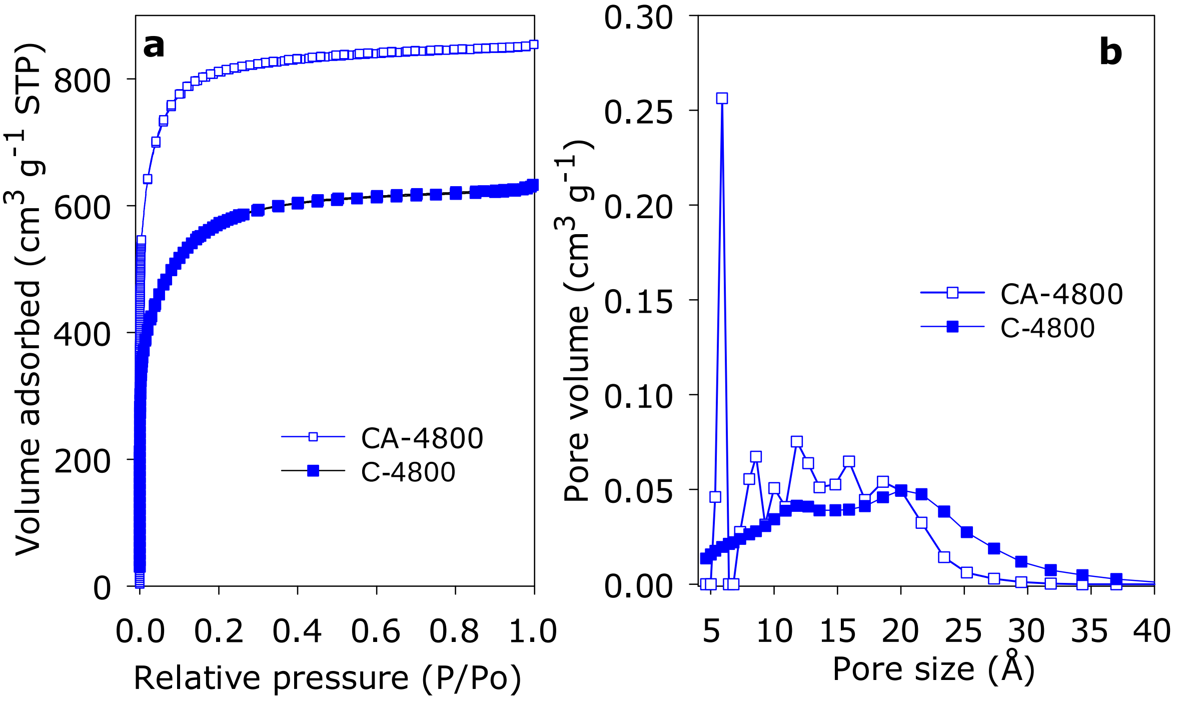

Supplementary Figure 6. Porosity of activated carbons prepared under similar conditions but from different precursors. (a) Nitrogen sorption isotherms and (b) pore size distribution curves of activated carbons prepared from cellulose acetate (CA-4800) or cellulose (C-4800) by activation of their respective hydrochars at 800 ^{o}C and KOH/hydrochar ratio of 4.

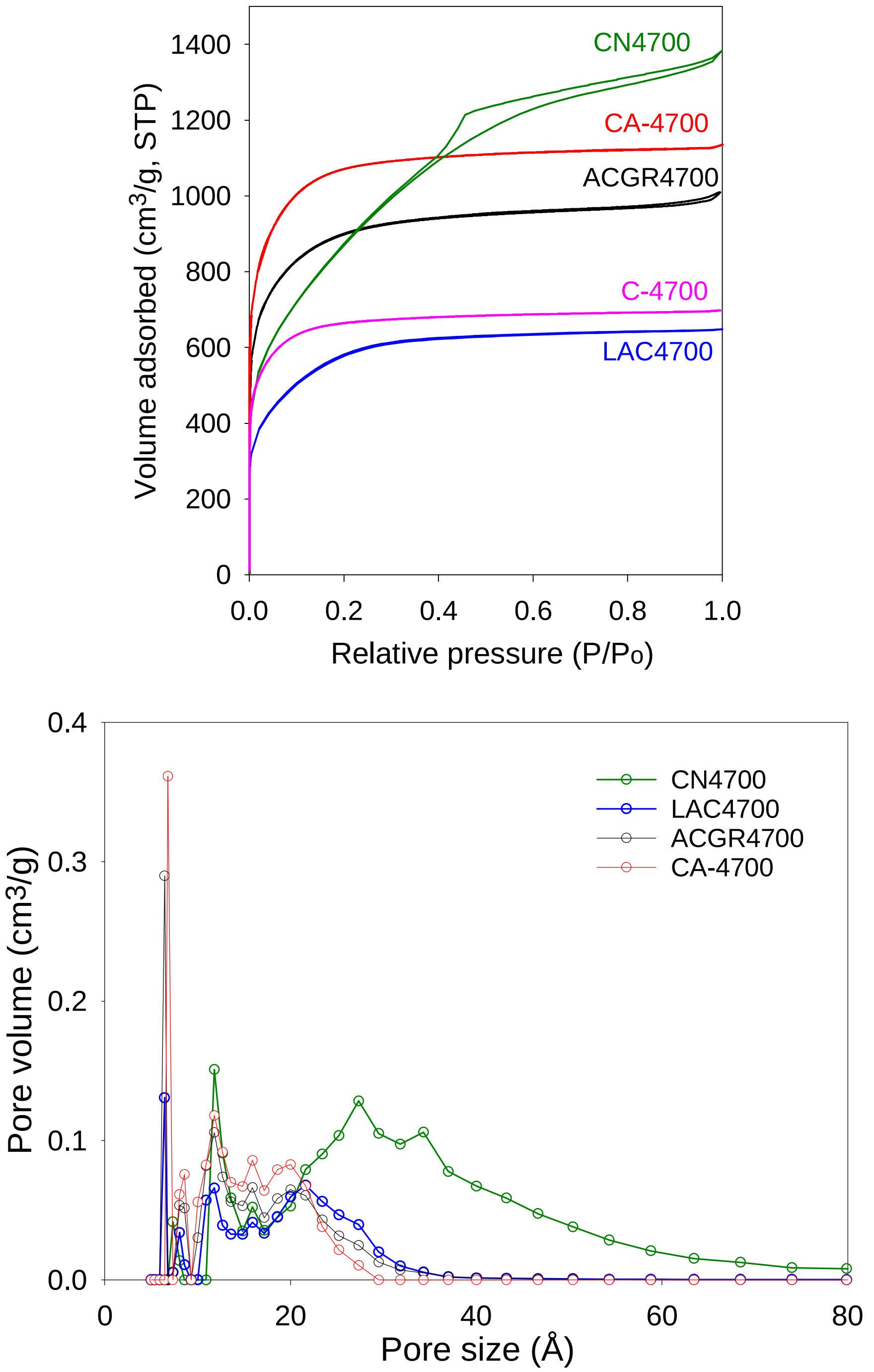

Supplementary Figure 7. Porosity of activated carbons prepared under similar conditions but from different precursors. Nitrogen sorption isotherms (top) and pore size distribution curves (bottom) of cellulose acetate-derived activated carbon (CA-4700) compared to analogous activated carbons derived from lignin (LAC4700), grass (ACGR4700) and carbon nanotube superstructures (CN4700) prepared under similar conditions (i.e., 700 ^{o}C and KOH/carbon ratio of 4).

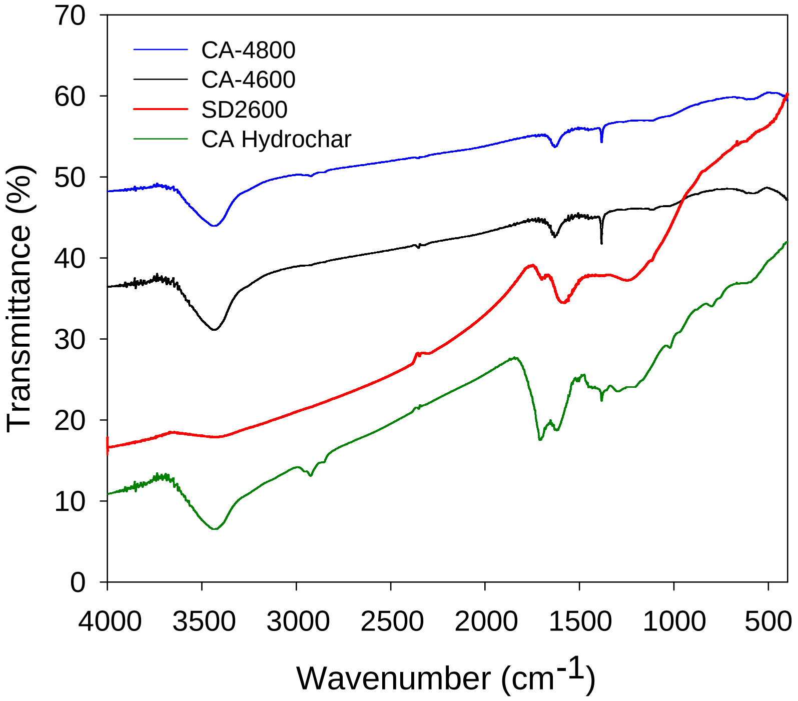

Supplementary Figure 8. IR spectra of cellulose acetate-derived CA-hydrochar and activated carbons derived from the CA-hydrochar compared to activated carbon derived from sawdust (SD2600). The IR spectra for SD2600 is adapted from reference 1.

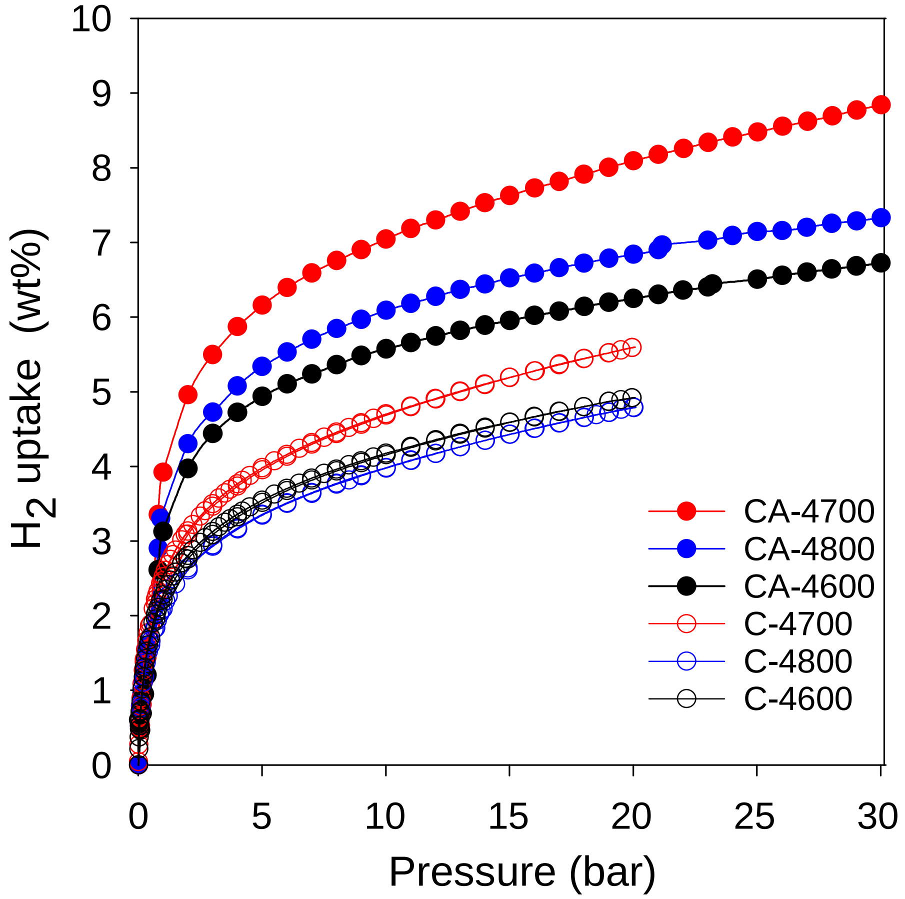

Supplementary Figure 9. Total hydrogen uptake of cellulose acetate-derived activated carbons (CA-4T) compared to cellulose-derived activated carbons (C-4T).

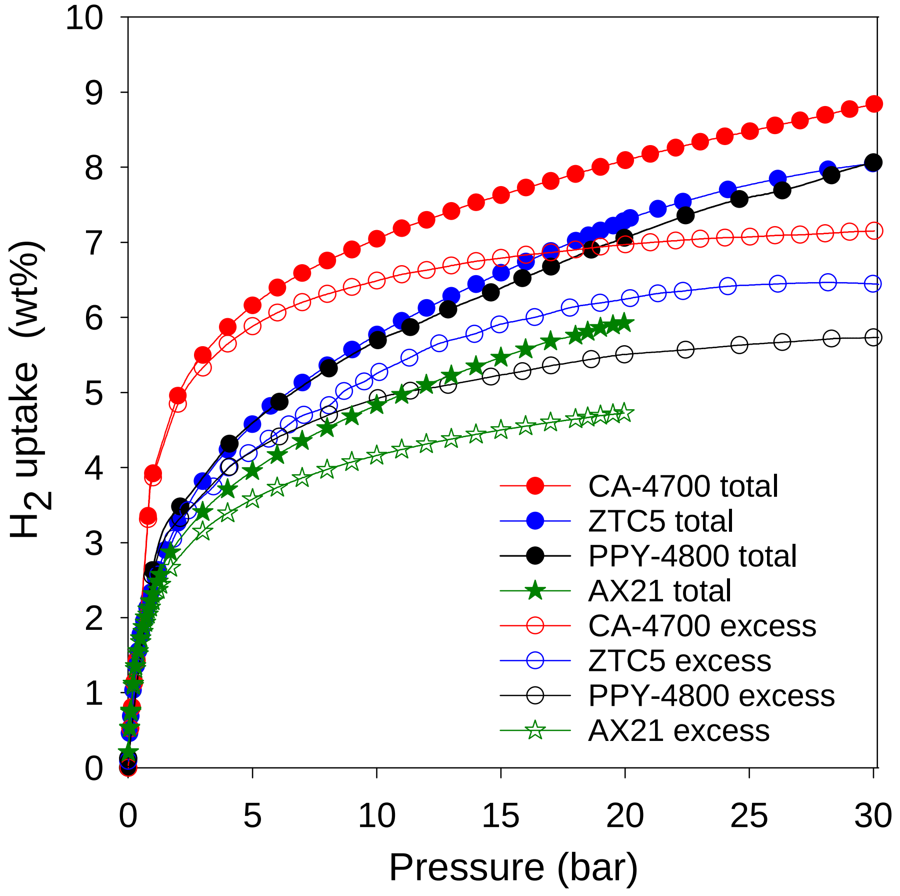

Supplementary Figure 10. Excess and total hydrogen uptake of activated carbons derived from cellulose acetate compared to benchmark polypyrrrole-derived compactivated carbon (PPY-4800), zeolite templated carbon (ZTC5) and commercially available activated carbon (AX21). Data for PPY-4800 and ZTC5 is taken from reference 2 and 3, respectively.

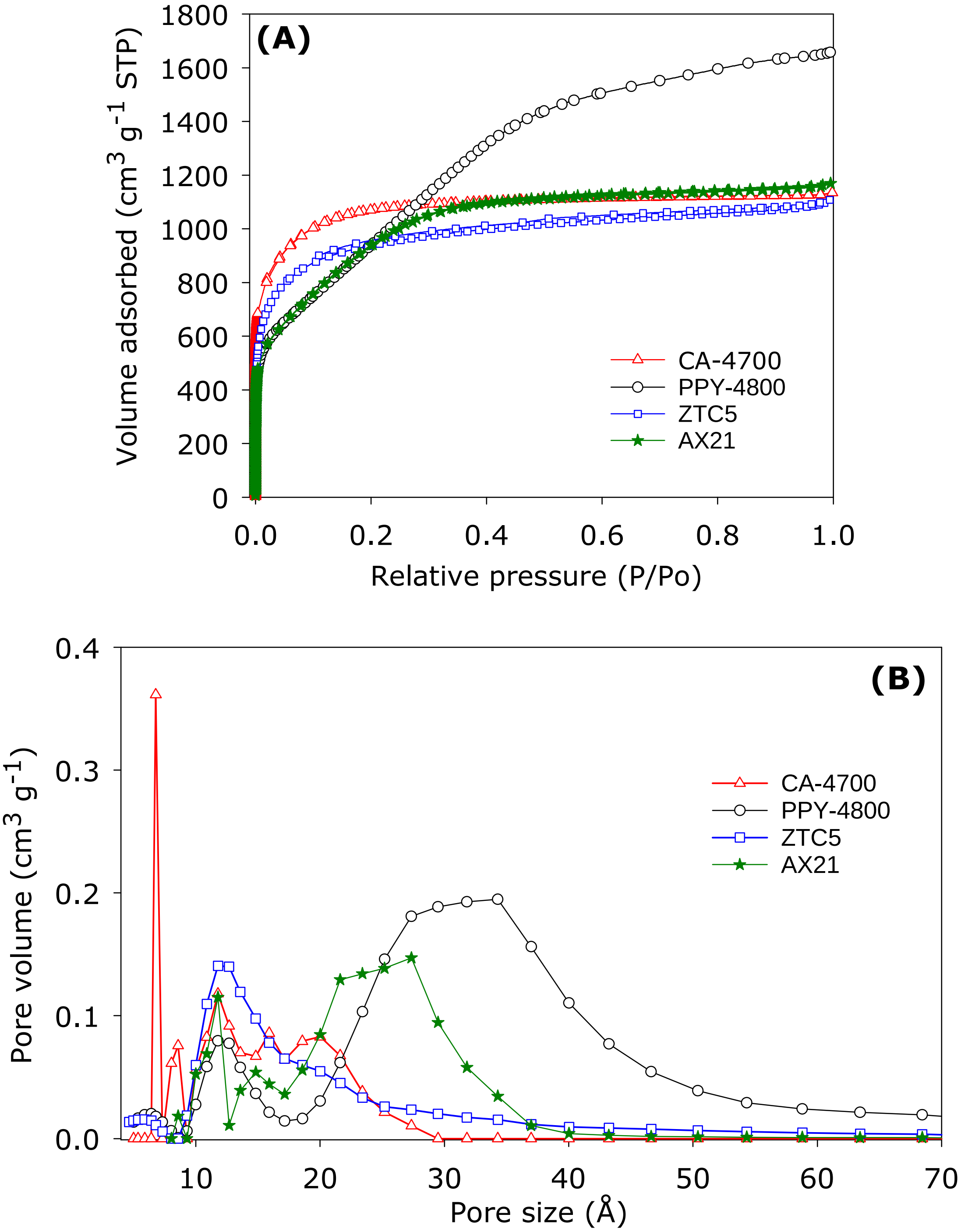

Supplementary Figure 11. Nitrogen sorption isotherms (A) and pore size distribution curves (B) of cellulose acetate-derived activated carbon (CA-4700) compared to to benchmark polypyrrrole-derived compactivated carbon (PPY-4800), zeolite templated carbon (ZTC-5) and commercially available activated carbon (AX21). Data for PPY-4800 and ZTC5 is taken from reference 2 and 3, respectively.

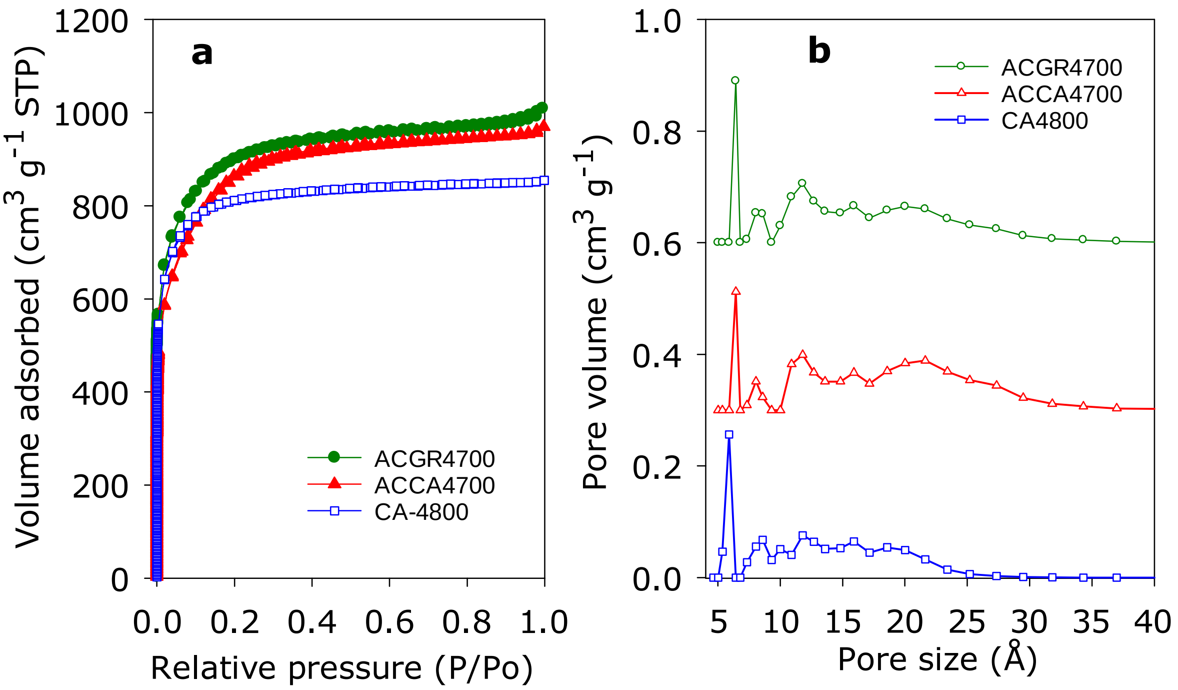

Supplementary Figure 12. Porosity of activated carbons prepared under varying conditions and from different precursors. (a) Nitrogen sorption isotherm and (b) pore size distribution curve of cellulose acetate-derived activated carbon (CA-4800) compared to analogous activated carbons with similar porosity derived from the hydrochar of Jujun grass (ACGR4700) and *Camellia Japonica* (ACCA4700). Data for samples ACGR4700) and ACCA4700 is taken from reference 4.

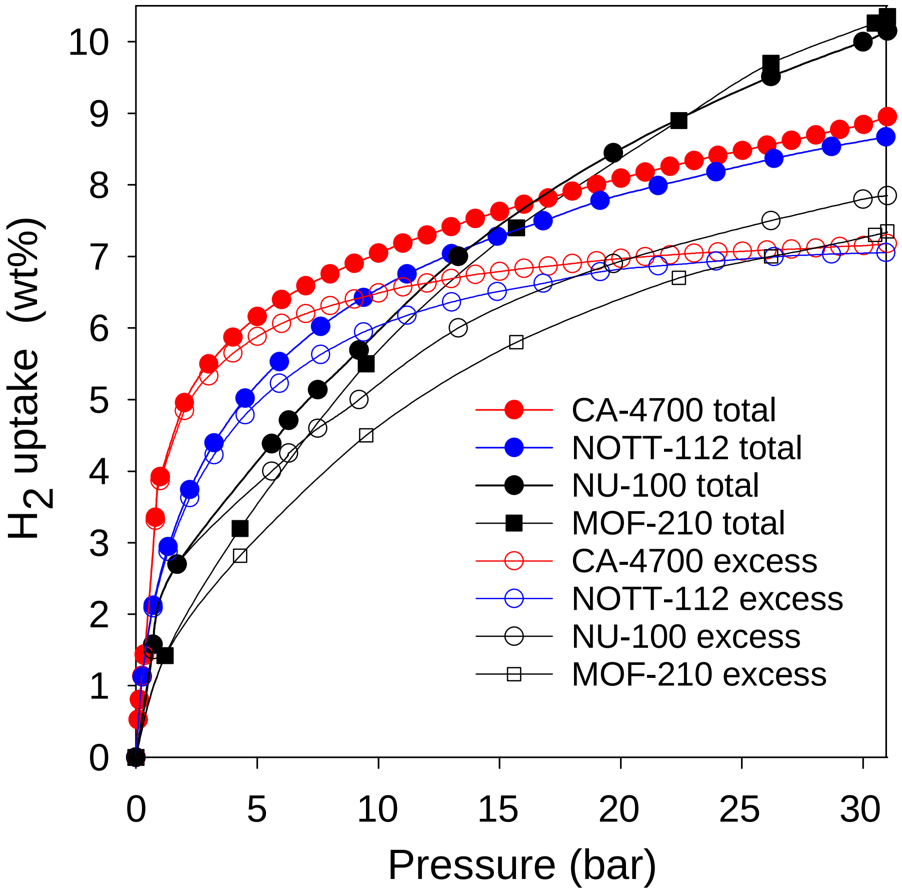

Supplementary Figure 13. Excess and total gravimetric hydrogen uptake of activated carbon (CA-4700) derived from cellulose acetate compared to the best benchmark high surface area metal organic frameworks (MOFs), namely, NOTT-112, NU-100 and MOF-210. Data for NOTT-112, NU-100 and MOF-210 is taken from reference 5, 6 and 7, respectively.

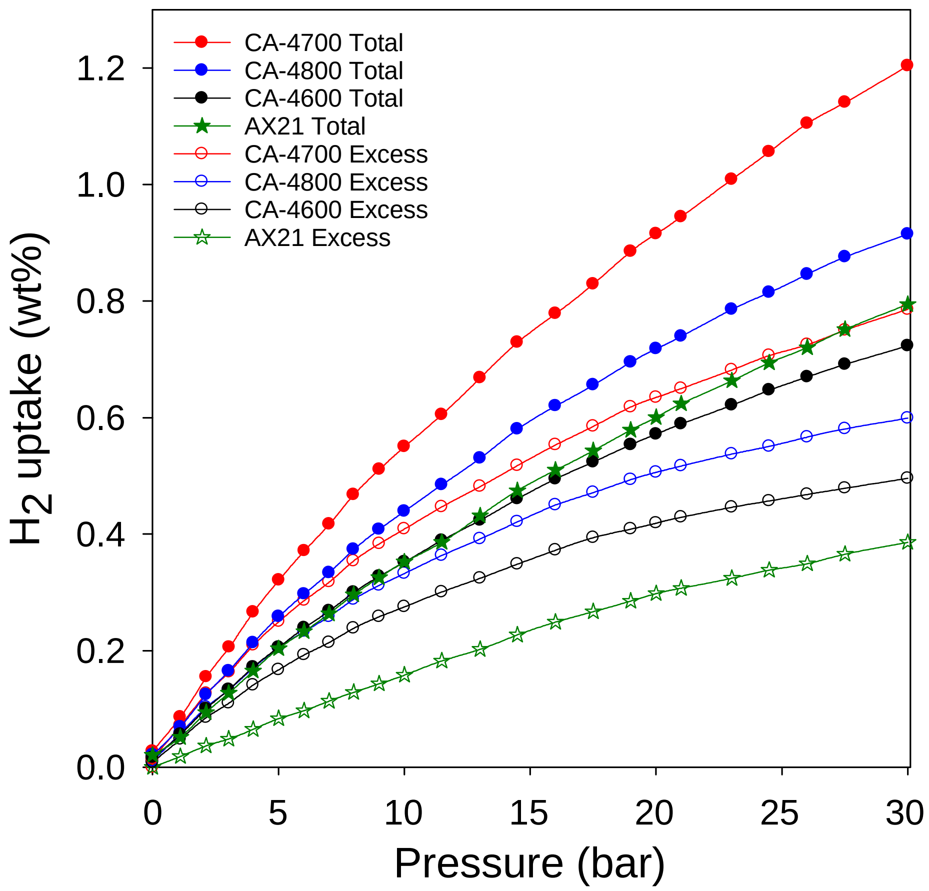

Supplementary Figure 14. Excess and total hydrogen uptake at 25 °C and pressure of up to 30 bar of CA-4T activated carbons derived from cellulose acetate and a commercially available activated carbon AX21.

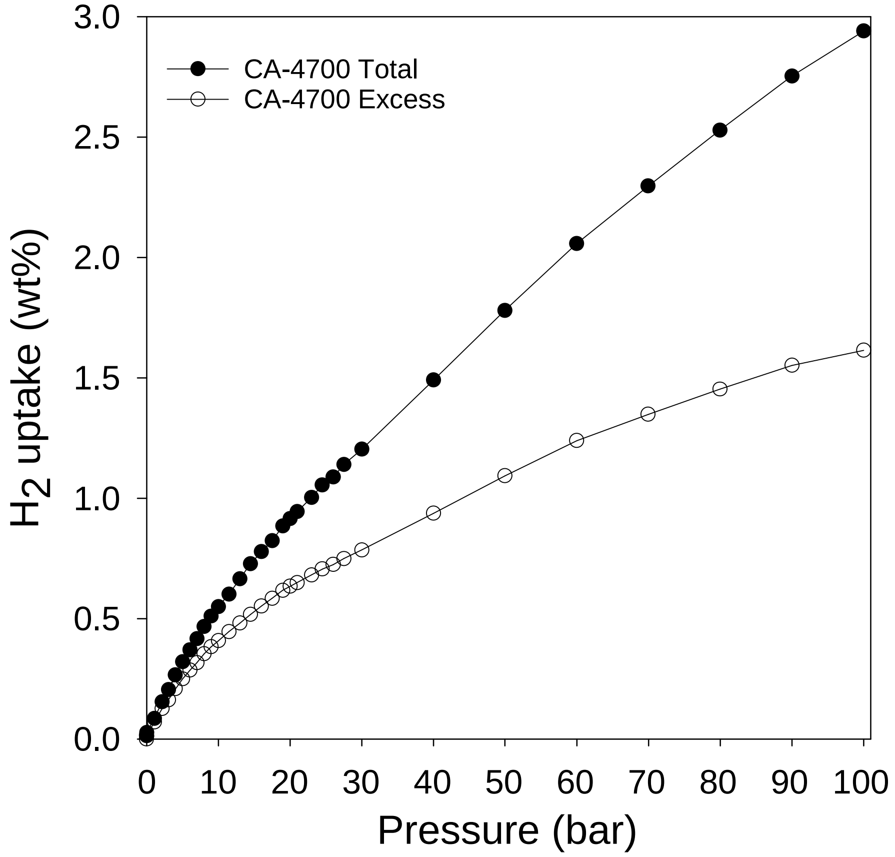

Supplementary Figure 15. Excess and total hydrogen uptake at 25 ºC and pressure of up to 100 bar of sample CA-4700.

Supplementary Table 1. Elemental analysis of cellulose acetate-derived CA-hydrochar and cellulose-derived C-hydrochar and activated carbons derived from the hydrochars.

Sample	C [%]	H [%]	O [%]	(O/C)[a]	(H/C)[a]
C-hydrochar	69.5	6.1	24.4	0.263	1.053
CA-hydrochar	66.2	3.9	29.9	0.339	0.707
C-4600	86.9	1.2	11.5	0.099	0.163
C-4700	92.2	0.6	7.1	0.057	0.072
C-4800	95.0	0.2	4.4	0.035	0.030
CA-4600	76.1	1.1	22.8	0.225	0.173
CA-4700	81.4	0.7	17.9	0.165	0.103
CA-4800	78.3	1.1	20.6	0.197	0.169

[a]Atomic ratio

Supplementary Table 2. Textural properties and H_2 uptake of cellulose acetate-derived activated carbons (CA-4T) compared to cellulose-derived activated carbons (C-4T).

Sample	Surface area[a] ($m^2\ g^{-1}$)	Pore volume[b] ($cm^3\ g^{-1}$)	Pore size[c] (Å)	H_2 uptake[d] (wt%)	
				1 bar	20 bar[e]
CA-4600	2001 (1790)	0.95 (0.79)	6/8.5/12	3.1	6.2 (5.6)
CA-4700	3771 (3484)	1.75 (1.54)	7/8.5/12	3.9	8.1 (7.0)
CA-4800	2864 (2662)	1.32 (1.17)	6/8.5/12	3.4	6.8 (6.0)
C-4600	2014 (1843)	0.94 (0.81)	12	2.3	4.9 (4.3)
C-4700	2370 (2201)	1.08 (0.96)	12/15	2.5	5.6 (4.9)
C-4800	2047 (1707)	0.98 (0.74)	12/20	2.1	4.8 (4.2)

Values in the parenthesis refer to: [a]micropore surface area and [b]micropore volume. [c]Pore size distribution maxima obtained from NLDFT analysis. [d]Gravimetric (wt%) H_2 uptake at -196 ^oC and various pressures (i.e., 1 or 20 bar).[e]The values in parenthesis are excess H_2 uptake.

Supplementary Table 3. Textural properties and H_2 uptake of cellulose acetate-derived activated carbon (CA-4700) compared to cellulose-derived activated carbon (C-4700), and analogous activated carbons derived from the hydrochar of other biomass, namely, glucose (G-4700), starch (S-4700), eucalyptus sawdust (E-4700), furfural (F-4700), lignin (LAC4700), jujun grass (ACGR4700), *Camellia Japonica* (ACCA4700), and carbon nanotube superstructures (CN4700), under similar activation conditions (i.e., at 700 ^oC and KOH/carbon ratio of 4).

Sample	Surface area[a] (m^2 g^{-1})	Pore volume[b] (cm^3 g^{-1})	Pore size[c] (Å)	H_2 uptake[d] (wt%)		Reference
				1 bar	20 bar[e]	
CA-4700	3771 (3484)	1.75 (1.54)	7/8.5/12	3.9	8.1 (7.0)	This work
C-4700	2370 (2201)	1.08 (0.96)	12/15	2.5	5.6 (4.9)	8
G-4700	2121 (2012)	1.00 (0.91)	12/15	2.4	5.3 (4.6)	8
S-4700	2194 (2082)	1.01 (0.92)	12/15	2.4	5.4 (4.7)	8
E-4700	2252 (2088)	1.03 (0.91)	12/15	2.5	5.6 (4.9)	8
F-4700	2179 (2067)	1.03 (0.94)	12/15	2.5	5.4 (4.7)	8
LAC4700	2038 (1832)	1.00 (0.84)	6/8/11/21	1.9	5.2 (4.4)	9
ACGR4700	3144 (2753)	1.56 (1.23)	6/8/12/20	2.4	5.5 (4.5)	4
ACCA4700	2983 (2500)	1.50 (1.14)	6/12/22	2.4	5.4 (4.5)	4
CN4700	3202 (1106)	2.14 (0.50)	8/12/28	2.4	6.7 (5.3)	10

Values in the parenthesis refer to: [a]micropore surface area and [b]micropore volume. [c]Pore size distribution maxima obtained from NLDFT analysis. [d]Gravimetric (wt%) H_2 uptake at -196 ^oC and various pressures (i.e., 1 or 20 bar).[e]The values in parenthesis are excess H_2 uptake.

Supplementary Table 4. Textural properties and H_2 uptake of cellulose acetate-derived activated carbon (CA-4700) compared to benchmark polypyrrrole-derived compactivated carbon (PPY-4800), zeolite templated carbon (ZTC-5) and commercially available high surface area activated carbon (AX21).

Sample	Surface area[a] (m^2 g^{-1})	Pore volume[b] (cm^3 g^{-1})	Pore size[c] (Å)	H_2 uptake[d] (wt%)		Reference
				1 bar	20 bar[e]	
CA-4700	3771 (3484)	1.75 (1.54)	7/8.5/12	3.9	8.1 (7.0)	This work
PPY-4800	3844 (-)	2.89 (-)	13/34	2.2	7.3 (5.5)	2
ZTC-5	3322 (2837)	1.66 (1.18)	12	2.5	7.3 (6.3)	3
AX21	3191 (1768)	1.80 (0.89)	12/21/25	2.1	5.9 (4.7)	This work

Values in the parenthesis refer to: **[a]**micropore surface area and **[b]**micropore volume. [c]Pore size distribution maxima obtained from NLDFT analysis. **[d]**Gravimetric (wt%) H_2 uptake at -196 ^oC and various pressures (i.e., 1 or 20 bar).[e]The values in parenthesis are excess H_2 uptake.

Supplementary Table 5. Excess H_2 uptake of cellulose acetate-derived activated carbon (CA-4800) compared to analogous activated carbons with similar porosity (i.e., surface area, pore volume, pore size and level of microporosity) derived from the hydrochar of Jujun grass (ACGR4700) and *Camellia Japonica* (ACCA4700).[4]

Sample	Surface area[a] (m^2 g^{-1})	Pore volume[b] (cm^3 g^{-1})	Pore size[c] (Å)	Elemental composition (wt%)			H_2 uptake[d] (wt%)	
				C [%]	H [%]	O [%]	1 bar	20 bar
CA-4800	2864 (2662)	1.32 (1.17)	6/8.5/12	78.3	1.1	20.6	3.4	6.0
ACGR4700	3144 (2753)	1.56 (1.23)	6/8/12	86.2	0.5	13.3	2.4	4.5
ACCA4700	2983 (2500)	1.50 (1.14)	7/8.5/12	87.0	0.5	12.5	2.4	4.5

The values in the parenthesis refer to: **[a]**micropore surface area and **[b]**micropore volume. **[c]**Pore size distribution maxima obtained from NLDFT analysis. **[d]**Excess (wt%) H_2 uptake at -196 ^oC and various pressures (i.e., 1 or 20 bar).

Supplementary Table 6. Surface area, pore volume hydrogen storage (at -196 ^oC and 20 or 30 bar) for CA-4T carbons compared to the best benchmark metal organic frameworks (MOFs).

Sample	Surface area ($m^2\ g^{-1}$)	Pore Volume ($cm^3\ g^{-1}$)	Gravimetric H_2 uptake (wt%)[a]		Volumetric H_2 uptake ($g\ l^{-1}$)[b]		Reference
			Excess	Total	Excess	Total	
CA-4600	2001	0.95	5.6 (5.8)	6.2 (6.7)	40 (41)	44 (48)	This work
CA-4700	3771	1.75	7.0 (7.2)	8.1 (8.9)	32 (33)	37 (41)	This work
CA-4800	2864	1.32	6.0 (6.1)	6.8 (7.3)	34 (34)	41 (41)	This work
NOTT-112[c]	3800	1.62	6.8 (7.0)	7.8 (8.6)	16 (17)	19 (21)	5,11
NU-100[c]	6143	2.82	6.9 (7.8)	8.5 (10.0)	11 (12)	13 (15)	6,11
MOF-210[c]	6240	3.60	6.4 (7.3)	8.4 (10.2)	8 (9)	11 (13)	7,11
MOF-210[d]	6240	3.60	6.4 (7.3)	8.4 (10.2)	16 (18)	22 (26)	7,11

[a]Gravimetric excess and total H_2 uptake at -196 ^oC and 20 bar or (for values in parenthesis) at 30 bar. [b]Volumetric excess and total H_2 uptake at -196 ^oC and 20 bar or (for values in parenthesis) at 30 bar. Packing density values ($g\ cm^{-3}$) used, according to reference 11, are: 0.23 (NOTT-112),[11] 0.15 (NU-100)[11] and 0.125 (MOF-210).[11] The packing density values are based on experimental data (reference 11) that shows that the actual "tapped density" of MOFs is roughly one-half of the 'ideal' crystal density. [d]Crystal density (0.25 $g\ cm^{-3}$)[7] was used to calculate volumetric storage capacity values.

Supplementary References

1. Balahmar, M., Mitchell, A. C. & Mokaya, R. Generalized mechanochemical synthesis of biomass-derived sustainable carbons for high performance CO_2 storage. *Adv. Energy Mater.* **5**, 1500867 (2015).
2. Adeniran, B. & Mokaya, R. Compactivation: A mechanochemical approach to carbons with superior porosity and exceptional performance for hydrogen and CO_2 storage. *Nano Energy* **16**, 173–185 (2015).
3. Masika, E. & Mokaya, R. Exceptional gravimetric and volumetric hydrogen storage for densified zeolite templated carbons with high mechanical stability. *Energy Environ. Sci*. **7**, 427–434 (2014).
4. Coromina, H. M., Walsh, D. A. & Mokaya, R. Biomass-derived activated carbon with simultaneously enhanced CO_2 uptake for both pre and post combustion capture applications. *J. Mater. Chem. A* **4**, 280–289 (2016).
5. Yan, Y., Lin, X., Yang, S., Blake, A. J., Dailly, A., Champness, N. R., Hubberstey, P. & Schroder, M. Exceptionally high H_2 storage by a metal organic polyhedral framework. *Chem. Commun*. 1025–1027 (2009).
6. Farha, O. K., Yazaydın, A. O., Eryazici, I., Malliakas, C. D., Hauser, B. G., Kanatzidis, M. G., Nguyen, S. T., Snurr, R. I. Q. & Hupp, J. T. De novo synthesis of a metal–organic framework material featuring ultrahigh surface area and gas storage capacities, *Nat. Chem*. **2**, 944–948 (2010).
7. Furukawa, H., Ko, N., Go, Y. B., Aratani, N., Choi, S. B., Choi, E., Yazaydin, A. O., Snurr, R. Q., O'Keeffe, M., Kim, J. & Yaghi, O.M. Ultra-high porosity in metal–organic frameworks. *Science* **329**, 424–428 (2010).
8. Sevilla, M., Fuertes, A. B. & Mokaya R. High density hydrogen storage in superactivated carbons from hydrothermally carbonized renewable organic materials. *Energy Environ. Sci*. **3**, 1400–1410 (2011).
9. Sangchoom, W. & Mokaya, R. Valorization of lignin waste: Carbons from hydrothermal carbonization of renewable lignin as superior sorbents for CO_2 and Hydrogen storage. *ACS Sust. Chem. Eng*. **3**, 1658–1667 (2015).
10. Adeniran, B. & Mokaya, R. Low temperature synthesized carbon nanotube superstructures with superior CO_2 and hydrogen storage capacity. *J. Mater. Chem. A* 3, 5148–5161 (2015).

11. Peng, Y., Krungleviciute, V., Eryazici, I., Hupp, J. T., Farha, O. K. & Yildirim, T. Methane storage in Metal–Organic Frameworks: Current records, surprise findings, and challenges. *J. Am. Chem. Soc*. 2013, **135**, 11887–11894.
